# Economic value of international missions and domestic initiatives to strengthen surgical care in low- and middle-income countries: systematic review

**DOI:** 10.1093/bjs/znaf207

**Published:** 2025-12-10

**Authors:** Martilord Ifeanyichi, Yannis Reissis, Rebecca Hakim, Maeve Bognini, Meskerem Kebede, Rachel Hargest, Rocco Friebel

**Affiliations:** Global Surgery Policy Unit, LSE Health, London School of Economics and Political Science, London, UK; Health Policy Research Group, Department of Pharmacology and Therapeutics, University of Nigeria Enugu Campus (UNEC), Enugu, Nigeria; Global Anaesthesia, Surgery, and Obstetric Collaboration (GASOC), Newcastle, UK; Global Surgery Policy Unit, LSE Health, London School of Economics and Political Science, London, UK; Global Anaesthesia, Surgery, and Obstetric Collaboration (GASOC), Newcastle, UK; Department of Surgery, University Hospitals Bristol and Weston NHS Foundation Trust, Bristol, UK; Global Surgery Policy Unit, LSE Health, London School of Economics and Political Science, London, UK; Global Surgery Policy Unit, LSE Health, London School of Economics and Political Science, London, UK; Global Surgery Policy Unit, LSE Health, London School of Economics and Political Science, London, UK; School of Medicine, University Hospital of Wales, Cardiff, UK; Global Surgery Policy Unit, LSE Health, London School of Economics and Political Science, London, UK; Department of Health Policy, London School of Economics and Political Science, London, UK; Center for Global Development Europe, London, UK

## Abstract

**Background:**

In many low- and middle-income countries (LMICs), domestic investments to strengthen surgical services compete with services delivered by international missions. While addressing the high burden of unmet surgical need is a priority, there remains limited evidence on the comparative economic value of different delivery options to guide investment decisions.

**Methods:**

Four databases and grey literature were searched for publications in any language from January 2013 to January 2023. Eligible studies evaluated the cost-effectiveness, cost-utility, or cost-benefit of international missions and domestic initiatives used for scale up of surgical care. Average cost-effectiveness ratios were computed for each intervention and then converted to 2022 international dollars (I$). Findings were synthesized narratively.

**Results:**

A total of 32 studies were identified (17 studies evaluated domestic surgical system strengthening programmes, 14 studies assessed international missions, and 1 study directly compared a domestic surgical development initiative against international missions). Financial protection schemes, investments in physical infrastructure, surgical residency training, and local missions were cost-effective, as were most of the international missions, compared with status quo or no intervention. However, when compared head-to-head, the unit costs per disability-adjusted life-year averted of domestic initiatives were significantly lower relative to the international missions—mean (standard deviation) I$27 051 (I$65 360) and median (interquartile range) I$498 (I$602) *versus* mean (standard deviation) I$515 500 (I$1 528 716) and median (interquartile range) I$5068 (I$31 618). The difference was statistically significant (Wilcoxon rank-sum test: *z* = 2.412; *P* = 0.016).

**Conclusion:**

Investments in domestic surgical system strengthening efforts provide better value for money than international missions and should be prioritized over international missions.

## Teaser

This systematic review compares the cost-effectiveness of international surgical missions and domestic initiatives to scale up surgical care in low- and middle-income countries (LMICs). While both approaches are generally cost-effective, domestic investments—in infrastructure, training, and financial protection for instance—offer significantly better value for money and should be prioritized in policy and planning.

## Introduction

In many low- and middle-income countries (LMICs), access to safe, timely, and affordable services has remained an exception, despite growing advocacy and evidence generation over the past decade^1^. The 2015 estimate of 5 billion people lacking access to surgical care^2^ has likely increased due to growth of the global population, particularly across LMICs, and the disruption to surgical care delivery caused by the COVID-19 pandemic and recent conflicts. Surgical need is further amplified by the effects of global warming^3^, adding to poor population health with harmful effects for economic development. Addressing surgical needs through integration of emergency, critical, and operative care, as well as system strengthening, as called for by the World Health Assembly Resolution 76.2, is a priority for state and non-state actors globally.

In addition to domestic interventions aiming at scaling up surgical care access and provision, which range from task-shifting initiatives to develop the surgical workforce^4^ to building mini hospitals^5–7^ or upgrading primary healthcare centres^8–10^, substantial volumes of surgical procedures in LMICs are delivered by international interventionist charities. The objective of charity interventions is to overcome the limited local surgical care supply, either through temporary platforms (most commonly in the form of short-term trips [missions] and rarely self-contained mobile structures like ships and aeroplanes) or by establishing permanent practices, such as surgical specialty hospitals^11^. Charitable organizations provide up to 55% of all surgical services in some LMICs^12^, contributing substantially to alleviation of surgical needs at a significant cost. Data from the USA suggest that 95 charitable organizations spent approximately US$2.53 billion providing surgical care in LMICs between 2007 and 2013^13^, whereas 160 charitable organizations from five high-income countries spent US$3.1 billion to deliver care across 15 surgical specialties between 2008 and 2013. These investments represent 11.5% of total global health expenditure over the interval^14^. While undoubtably impactful for individual patients, it is possible that international missions may undermine local efforts in system strengthening, effectively removing accountability from governments to provide domestic investments for services to address local needs^15^.

Investments in surgical care strengthening are limited, providing incentives for value maximization in the quest for societal welfare. This includes integrating evidence from economic evaluations on the comparative analysis of costs and consequences related to alternative interventions into priority setting and system planning^16^. Previous research has provided conflicting evidence on the cost-effectiveness of missions, particularly when compared with competing, domestically available service delivery platforms^11,17^. As LMICs move towards the adoption of universal healthcare (UHC) benefit packages that include surgical care components, there remains a critical evidence gap to guide the adoption of surgical service delivery in this complex interplay of domestic and international initiatives. This systematic literature review aimed to synthesize and compare the evidence on the cost-effectiveness of domestic investments in surgical capacity *versus* international missions.

## Methods

### Search strategy and information sources

This systematic review was conducted in accordance with the guidelines of the Centre for Reviews and Dissemination^18^ and the *Cochrane Handbook for Systematic Reviews of Interventions*^19^. A broad search strategy designed based on three overarching search blocks—‘surgery and anaesthesia’, ‘cost-effectiveness’, and ‘LMICs’—was adopted. The primary strategy was designed on Ovid MEDLINE and then adapted to Embase, Global Health, and EconLit (*Table S1*). The review comprised all literature published between January 2013 and January 2023. A grey literature search was conducted, including a reference review and website searches (for example WHO and World Bank websites). No language restrictions were applied. Conference presentations, abstracts, dissertations, and animal studies were excluded.

### Selection procedures

Deduplication was performed in EndNote. Screening of titles and abstracts (conducted in Rayyan—a web and mobile app designed for systematic reviews^20^) and full-text review were performed in parallel by two researchers. Conflicts between reviewers were addressed via discussion and resolved by a third reviewer when consensus was not reached. Inclusion and exclusion of articles was based on pre-specified eligibility criteria, in line with the Population, Intervention, Comparator, Outcome, and Study Design (PICOS) framework^21^. Studies that conducted cost-effectiveness analysis (CEA), cost-utility analysis (CUA), or cost-benefit analysis (CBA) of interventions in LMICs (defined according to World Bank classification) were included. Studies evaluating policies, programmes, and investments—whether empirical or hypothetical—aimed at improving supply and/or demand for surgery were included. Evaluations of specific surgical procedures were excluded. The complete list of applied eligibility criteria is available in *Table S2*.

### Data extraction

Two reviewers performed the data extraction using a customized, pretested data collection form in Microsoft Excel. For each article, one researcher conducted the data extraction, while another researcher independently reviewed the data extraction to check the completeness and accuracy of all data. A 10% sample was assessed by a third reviewer for verification purposes. Researchers extracted information on study characteristics, including bibliographic information, intervention, study methodologies, and all empirical evidence related to cost-effectiveness. The Drummond checklist was employed to assess the quality of the studies^21^.

### Data analysis, synthesis, and reporting

A descriptive analysis of study characteristics and results was performed for all included studies. To facilitate comparison across years and countries, the incremental cost-effectiveness ratios (ICERs) were converted from the original years to 2022 equivalents using World Bank gross domestic product (GDP) deflators^22^ and then to international dollars (I$) using World Bank purchasing power parities (PPPs)^23^. For studies without information on the currency year, the publication year was assumed to be the currency year.

While ICERs permit comparisons between pairs of alternative interventions, they do not allow meaningful one-on-one comparison between otherwise disparate interventions (for example a mission trip and a local investment). To conduct such comparisons, average cost-effectiveness ratios (ACERs) were computed for each intervention by dividing the total cost associated with the intervention by the total benefit. This approach has been previously applied when comparing the cost-effectiveness of surgical procedures with the cost-effectiveness of conventional public health programmes^24,25^. All ACERs were converted to 2022 I$. Although studies differed in design, it was assumed that variations in design across the international missions and local interventions were random and, as such, variations in the costs per benefit across the two groups would represent the actual variations in cost-efficiency across the two groups. However, this analysis was restricted to only a subset of studies that reported the benefits in quality-adjusted life-years (QALYs) gained or disability-adjusted life-years (DALYs) averted, with the implicit assumption that DALYs and QALYs wield equal economic value^24,25^. Also, interventions that contained subcomponents not directly related to access to surgery (for example ‘mainstream education’^26^) were excluded. Mean (standard deviation) and median (interquartile range) values were computed for both groups and compared using a two-sample Wilcoxon rank-sum (Mann–Whitney) test.

Due to heterogeneities in study population characteristics and modelling approaches (for example types of costs included), full or advanced meta-analyses were not conducted. A narrative synthesis was used to present the results of this study, in accordance with the Preferred Reporting Items for Systematic Reviews and Meta-analyses (PRISMA)^27^.

This review was registered with Open Science Framework Registries (https://doi.org/10.17605/OSF.IO/V6SX2).

## Results

**Fig. 1:**
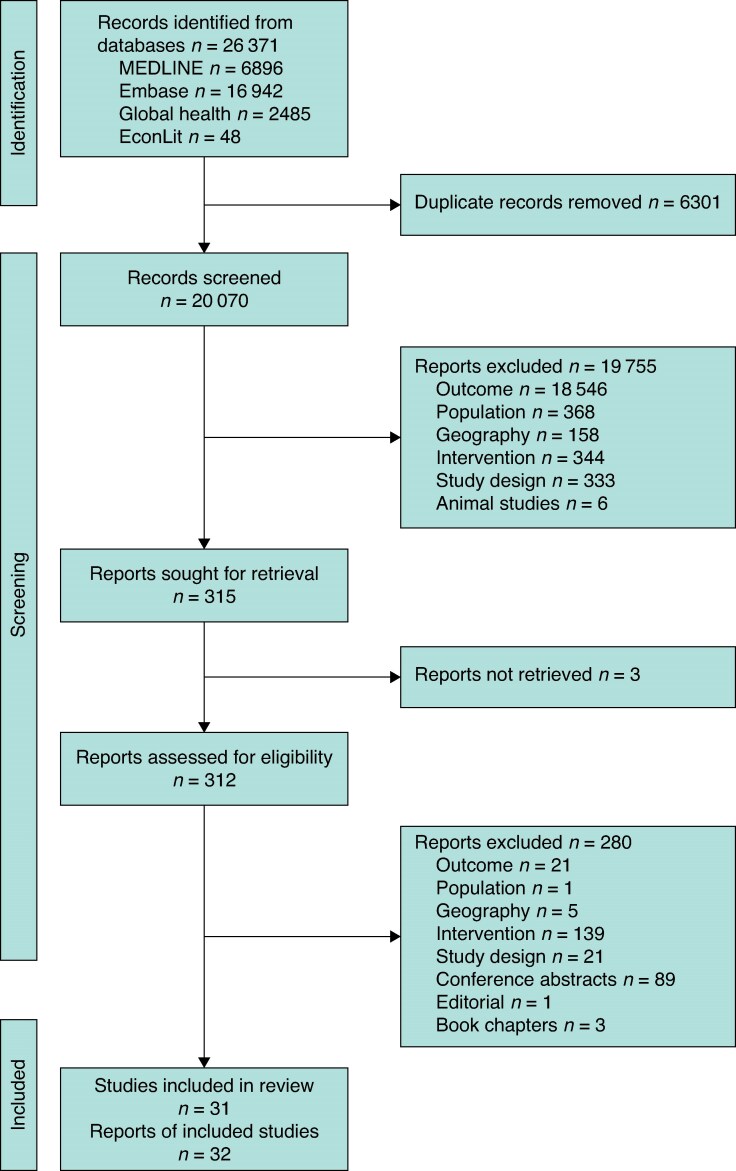
PRISMA flow diagram

A total of 26 371 articles were identified, including 20 070 after deduplication. A total of 315 articles underwent full-text review, with a total of 32 unique studies meeting the inclusion criteria (see *Fig. 1*).

### Study characteristics

All included studies were published in English. Seventeen studies evaluated local or domestic initiatives (see *Table 1*), with the majority of these involving hypothetical scenarios of scale up of specific procedures (9 studies); others covered interventions towards financial protection (3 studies), physical infrastructure (3 studies), training programmes (1 study), and local mission trips (1 study). Fourteen studies assessed the cost-effectiveness of mission trips undertaken by international non-governmental organizations (NGOs) (see *Table 2*). One study directly compared investment in domestic capacity *versus* international missions. Most studies (23 studies) performed a CUA, often reporting benefits as DALYs averted; 78% of all studies reported costs in United States dollars (US$). Studies spanned a wide range of specialties, with nine relating to more than one specialty. Among those that focused on single specialties, the most studied was paediatric surgery (4 studies), followed by otorhinolaryngology (3 studies) and plastic surgery (3 studies). The geographical distribution included sub-Saharan Africa (28 studies) and Latin America and the Caribbean (26 studies). Further details of the characteristics and results of the included studies are available in *Table S3*.

**Table 1:** Overview of economic evaluation of domestic interventions

First author, year Country	Type of economic evaluation	Intervention	Surgeries	Comparator	Perspective	Discount (effect)	Discount (costs)	ICER in I$	Threshold	Conclusion
**Scale up of national surgical programmes**										
Emmett, 2019^26^	CUA	Cochlear implantation + mainstream education	Cochlear implantation	Deaf education with sign language	Societal	3%	3%	Cost per DALY averted	ICER < 3×GDP per capita	
Nepal								$210 094		Not cost-effective
Bangladesh								$35 623		Cost-effective
Cambodia								$228 484		Cost-effective
Pakistan								$214 883		Not cost-effective
India								$173 466		Cost-effective
Philippines								$225 987		Cost-effective
Indonesia								$256 719		Cost-effective
Sri Lanka								$258 687		Cost-effective
Emmett, 2015^28^	CUA	Cochlear implantation + mainstream education	Cochlear implantation	Deaf education with sign language	Societal	3%	3%	Cost per DALY averted	ICER < 3×GDP per capita	
South Africa								−$30 179		Cost-effective
Nigeria								$96 757		Cost-effective
Kenya								$130 530		Not cost-effective
Rwanda								$94 180		Not cost-effective
Uganda								$89 978		Not cost-effective
Malawi								$110 019		Not cost-effective
Emmett, 2016^29^	CUA	Cochlear implantation + mainstream education	Cochlear implantation	Deaf education with sign language	Societal	3%	3%	Cost per DALY averted	ICER < 3×GDP per capita	
Brazil								$76 201		Very cost-effective
Colombia								$274 725		Very cost-effective
Ecuador								$10 043 263 300		Cost-effective
Guatemala								$370 216		Cost-effective
Paraguay								$287 233		Cost-effective
Trinidad and Tobago								$62 260		Very cost-effective
Venezuela								$116 724*		Cost-effective
Bansi-Matharu, 2023^30^	CUA	Circumcision for those aged 15–64 years for 5 years (modelled over 50 years; model names provided below)	Circumcision	No further circumcision	Healthcare provider	3%	3%	Cost per DALY averted (per 10 million men)	$500 per DALY averted	
South Africa		Goals-ASM						$335 674		Cost-effective
		Optima HIV						$247 442		Cost-effective
		EMOD						$22 987		Cost-effective
		Thembisa						$436 511		Cost-effective
Malawi		Goals-ASM						$92 597		Cost-effective
		Optima HIV						$269 927		Cost-effective
Zimbabwe		Goals-ASM						$52 215		Cost-effective
		Optima HIV						$235 599		Not cost-effective
Haacker, 2016^31^ South Africa	CEA	Voluntary male circumcision using an adapted version of the ASSA2008 HIV model	Circumcision	No intervention	Health system	–	5%	Cost per HIV infection averted: $561 (at birth)–$56 713 (at 55 years)	–	Very effective at reducing HIV incidence and cost of treatment, especially when performed between 20 and 25 years
Holmes, 2021^32^ South Africa	CBA	Targeting strategy for uncircumcised men in the Gauteng province	Circumcision	Status quo (routine outreach strategies)	Healthcare provider	–	3%	Cost per 100 men enrolled: $1493	–	More cost-effective than status quo
Bayani, 2021^33^ Philippines	CUA	Three scenarios for renal replacement therapy Adequate haemodialysis (PhilHealth covers three sessions/week and costs of immune suppression) Peritoneal dialysis first for all eligible patients Peritoneal dialysis first and pre-emptive transplant	Peritoneal dialysis catheter Kidney transplant	Status quo (94% on haemodialysis, 4% on peritoneal dialysis, and 2% receive transplant; immune suppression not covered by PhilHealth)	Societal	3%	3%	Cost per QALY gained $88 781 $33 705 $33 241	$8747 per QALY gained	Not cost-effective for any scenario A move towards peritoneal dialysis first with early transplant would be more cost-effective than the status quo
Nunes, 2018^34^ Cape Verde	CBA	Scenario whereby Cape Verde performs hip replacements for both fractures and arthritis	Total hip replacement	Status quo (hip fractures are treated in Cape Verde and hip arthritis in Portugal)	Societal	–	–	Net monetary benefit: $1 826 701 ($1353 per patient over 6 years)	–	Significant return on investment
Le, 2016^35^ India	CUA	Cataract surgeries performed in the Avarind Eye Care System	Cataract removal	No surgery	Healthcare provider	–	10%	Cost per QALY gained: $783	ICER < GDP per capita	Cost-effective
**Financial protection policies**										
Shrime, 2016^36^ Ethiopia	CEA	Six scenarios to cover nine surgeries in rural Ethiopia Universal public finance Universal public finance + vouchers Task sharing Universal public finance + task sharing Universal public finance + task sharing + vouchers Task sharing + vouchers	Caesarean section Vacuum aspiration Hysterectomy Appendicectomy Salpingectomy Laparotomy Limb traction Chest tube Amputation	Status quo (majority of surgeries are performed in urban areas)	Societal	–	–	Cases of poverty averted; number of deaths averted (per 1 million people) for every US$100 000 spent 38; 2 48; 1 +145 cases of poverty; 64 +10 cases of poverty; 12 27; 3 +12 cases of poverty; 9	–	Distribution of benefits between wealth quintiles is most equitable when non-medical costs are covered
Verguet, 2015^37^ Ethiopia	CEA	Universal public financing for nine interventions (treatment for diarrhoea, malaria, pneumonia, hypertension, and TB, measles vaccination, rotavirus vaccination, pneumococcal conjugate vaccination, and caesarean section surgery)	Caesarean section	Eight primary care interventions	Societal	–	–	Cases of poverty averted; number of deaths averted per US$100 000 spent (values shown for caesarean section only) 98; 141	–	Funding for caesarean section would avert the most impoverishment and the third highest number of deaths
Essue, 2020^38^ Vietnam	CEA	Two scenarios for cataract surgery Remove medical out-of-pocket costs Remove medical and non-medical out-of-pocket costs	Cataract removal	Status quo (patients pay all out-of-pocket costs)	Societal	3%	3%	Cost per DALY averted $3547 (males); $4146 (females) $7006 (males); $8166 (females)	–	Addressing both medical and non-medical out-of-pocket costs increased protection for the poorest patients, especially females
**Hypothetical scale up of infrastructure**										
Watkins, 2016^39^ Hypothetical low-income African country	CUA	Two policy scenarios for rheumatic heart disease Build a surgical centre Refer for surgery abroad	Heart valve repair/replacement	Scale up primary and secondary prevention	Health system	3%	3%	Cost per DALY averted $23 827 $3814	ICER < 3×GDP per capita	Not cost-effective Cost-effective
Yap, 2021^40^ Uganda	CUA	Build a dedicated paediatric theatre	Variety of emergency, elective, and cancer operations	No theatre	Societal	–	–	$43	ICER < 3×GDP per capita	Cost-effective
Yap, 2018^41^ Uganda	CUA	Build a dedicated paediatric theatre	A variety of emergency, elective, and cancer operations	No theatre	Healthcare provider	3%	3%	Cost per DALY averted: $21	ICER < 3×GDP per capita	Very cost-effective
**Training programmes**										
Agwu, 2021^42^ Malawi	CUA	5 years of orthopaedic residency training	Fracture management	No surgical training	Public health sector	3%	3%	Cost per DALY averted: $591	ICER < GDP per capita	Very cost-effective
**Domestic mission trips**										
Gyedu, 2017^43^ Ghana	CUA	All outreach trips by the AMOG group between 2011 and 2016	Variety of general, orthopaedic, urological, plastic, and obstetric surgeries	No surgery	Societal	–	–	Cost per DALY averted: $404	ICER < GDP per capita	Very cost-effective

^*^Value not adjusted using PPP due to unavailability of relevant conversion factor. ICER, incremental cost-effectiveness ratio; I$, international dollars; CEA, cost-effectiveness analysis; TB, tuberculosis; DALY, disability-adjusted life-year; CUA, cost-utility analysis; GDP, gross domestic product; HIV, human immunodeficiency virus; CBA, cost-benefit analysis; QALY, quality-adjusted life-years; AMOG, ApriDec Medical Outreach Group; PPP, purchasing power parity.

**Table 2:** Overview of economic evaluation of international missions

First author, year Country NGO	Type of economic evaluation	Intervention/types of surgery	Comparator	Perspective	Discount (effect)	Discount (costs)	ICER in I$	Threshold	Conclusion
Dolan, 2021^44^ St Vincent and the Grenadines World Paediatric Project (USA)	CUA	914 paediatric surgeries between 2002 and 2019	No surgery	Healthcare provider	3%	3%	Cost per DALY averted: $5068 (average)	ICER < 50% GDP per capita	Cost-effective
		Ophthalmology					$3228		Cost-effective
		Orthopaedics					$19 115		Not cost-effective
		Plastics					$4808		Cost-effective
		General Surgery					$2683		Cost-effective
		Urology					$14 823		Not cost-effective
		Neurosurgery					$2808		Cost-effective
Goldfarb, 2023^45^ St Vincent and the Grenadines World Paediatric Project (USA)	CUA	3 mission trips for paediatric upper limb surgeries between 2016 and 2019	No surgery	Healthcare provider	–	–	Cost per DALY averted: $13 330	ICER < GDP per capita	Cost-effective
Cardarelli, 2018^46^ China, Macedonia, Honduras, Iran, Iraq, Libya, Nigeria, Pakistan, Russia, and Ukraine William Novick Cardiac Alliance (USA)	CUA	470 paediatric cardiac surgeries performed in 2015	No surgery	Societal	–	–	Cost per DALY averted: $171	ICER < GNI	Very cost-effective
Davis, 2014^47^ Guatemala University of Michigan (USA)	CUA	17 paediatric neurosurgical operations performed during the 2014 mission trip	No intervention	Societal	3%	3%	Cost per DALY averted: $1030	ICER < GDP per capita	Cost-effective
Billig, 2020^48^ Bolivia, Ethiopia, Vietnam, Zimbabwe, St Vincent, Guatemala, Trinidad and Tobago, and Ghana The Touching Hands Project, ReSurge International (USA)	CBA	15 hand surgery mission trips between 2015 and 2018	No surgery	Societal	3%	3%	Net economic benefit Human capital approach: $238 456 Value of a statistical life-year approach: $576 716	–	Significant return on investment Greatest economic benefit from trips that emphasized education/training of local personnel
Qiu, 2019^49^ Bolivia, Ethiopia, Vietnam, Zimbabwe, St Vincent, Guatemala, and Trinidad and Tobago The Touching Hands Project, ReSurge International (USA)	CUA	14 hand surgery mission trips between 2015 and 2018	No surgery	Healthcare provider	3%	3%	Cost per DALY averted: $631	ICER < 3×GDP per capita	Very cost-effective
Tadisina, 2014^50^ Honduras Ruth Paz Foundation (USA)	CUA	Hand surgery mission trip in 2006	No surgery	Healthcare provider	–	–	Cost per DALY averted: $1713	ICER < 2×GNI per capita	Cost-effective
Taylor, 2021^51^ Nicaragua Esperanca (USA)	CUA	16 mission trips between 2006 and 2014	No surgery	Healthcare provider	3%	3%	Cost per DALY averted	ICER < 3×GDP per capita	
		General Surgery					$8591		Cost-effective
		Paediatric Surgery					$4907		Very cost-effective
		Gynaecology					$5745		Very cost-effective
		Orthopaedics					$20 519		Cost-effective
Shillcutt, 2013^52^ Ecuador Operation Hernia (UK)	CUA	102 inguinal hernia repairs performed during two trips to a rural hospital in 2010	No surgery	Healthcare provider	3%	3%	Cost per DALY averted: $4 861 461	ICER < GNI per capita	Cost-effective
Ament, 2014^53^ Bolivia Solidarity Bridge (USA)	CUA	16 spinal surgeries performed between 2008 and 2011	No surgery	Societal	3%	3%	Cost per QALY gained $39 315	$55 906 per QALY gained	Cost-effective
*Hackenberg, 2015^54^ India Operation Smile (UK)	CUA	Two strategies for cleft lip and palate repair between 2006 and 2012 17 mission trips Comprehensive care centre	No surgery	Healthcare provider	–	–	Cost per DALY averted $605 $247	–	Both cost-effective
Hamze, 2017^55^ Burundi, Democratic Republic of Congo, Ethiopia, Kenya, Rwanda, South Sudan, Tanzania, and Uganda Smile Train (USA)	CUA	37 274 cleft lip and palate repairs between 2006 and 2014	No surgery	Healthcare provider	3%	–	Cost per DALY averted: $63	ICER < GDP per capita	Very cost-effective
Egle, 2014^56^ Dominican Republic Midwest Medical Missions Michigan Chapter (USA)	CUA	General and gynaecological surgeries performed during two trips between 2010 and 2012	No surgery (also compared with equivalent surgeries at host institution)	Healthcare provider	–	–	Cost per DALY averted $785	ICER < GDP per capita	Very cost-effective (79% cheaper than equivalent surgeries at host institution)
Schlegelmilch, 2017^57^ Ecuador CAMTA (Canada)	CUA	157 total hip replacements between 2007 and 2011	No surgery	Healthcare provider	3.5%	–	Cost per QALY gained	ICER < GDP per capita	
		Unilateral					$340 748 920		Not cost-effective
		Bilateral					$225 451 250		Very cost-effective
		Staged (two surgeries in different years)					$336 872 632		Not cost-effective (All cost-effective according to NICE guidelines)
Eblovi, 2019^58^ Honduras Variety of volunteers from HICs	CUA	Mixture of general, urological, orthopaedic, ENT, ophthalmological, and gynaecological surgeries performed during trips to a rural ambulatory centre in 2017	No surgery	Societal	3%	3%	Cost per DALY averted: $1674	ICER < GDP per capita	Cost-effective

*Compares international missions *versus* a domestic surgical system investment. NGO, non-governmental organization; ICER, incremental cost-effectiveness ratio; I$, international dollars; CUA, cost-utility analysis; DALY, disability-adjusted life-year; GDP, gross domestic product; GNI, gross national income; CBA, cost-benefit analysis; QALY, quality-adjusted life-year; CAMTA, Canadian Association of Medical Teams Abroad; NICE, National Institute for Health and Care Excellence; HICs, high-income countries; ENT, ear/nose/throat.

### Domestic interventions

Verguet *et al*.^37^ (2015) showed that caesarean section averted the most cases of poverty (98 cases averted per US$100 000 spent) in a scenario of universal public financing for a bundle of nine interventions (measles vaccination, rotavirus vaccination, pneumococcal conjugate vaccination, diarrhoea treatment, malaria treatment, pneumonia treatment, caesarean section surgery, hypertension treatment, and tuberculosis treatment) in Ethiopia. Shrime *et al*.^36^ (2016) and Essue *et al*.^38^ (2020) demonstrated that combined policies such as free surgery, transportation vouchers, and task sharing had the greatest financial impact in Ethiopia and Vietnam.

National procedure-specific scale-up programmes showed variable cost-effectiveness by region. Investments in cochlear implantation were cost-effective in Latin America^29^ and South-East Asia^26^, but not in most of the sub-Saharan African countries (4 of 6) due to lower GDP per capita^28^. Voluntary circumcision scale-up programmes in South Africa showed substantial return on investment and human immunodeficiency virus (HIV) reductions^30–32^. Similarly, shifting from haemodialysis to peritoneal dialysis in the Philippines^33^ and local (in-country) hip replacement surgery for hip arthritis in Cape Verde (as opposed to treatment overseas) demonstrated significant cost savings^34^.

Locally led surgical outreach missions, such as those by the ApriDec Medical Outreach Group in Ghana, were highly cost-effective, with costs of I$404 per DALY averted^43^. Providing surgical services through wholly local surgical mission trips to underserved communities might be a viable option for countries working to reduce the burden of surgical morbidity in their populations.

Watkins *et al*.^39^ (2016) modelled the cost of building a surgical centre in a hypothetical low-income African country to treat rheumatic heart valve disease. Due to the high costs of cardiac surgery, this approach would result in a high cost per DALY averted, when compared with scaling up primary and secondary prevention, arguing in favour of referring for surgery abroad instead. In contrast, Yap *et al*.^41^ (2018) reported a very low cost per DALY averted for building a dedicated paediatric operating room in Uganda.

Investments in training programmes are cost-effective. Agwu *et al*.^42^ (2021) reported that orthopaedic surgical residency training in Malawi had an ICER of I$591 per DALY averted. Over a 35-year career, it would cost an average of US$448 600 to train and compensate a residency-trained surgeon, who would avert approximately 5570 DALYs performing fractures surgeries over the same interval.

### International mission trips

All studies comparing surgeries performed by international NGOs with no surgery found the missions to be cost-effective, though thresholds varied. Most NGOs originated from North America, targeting Latin America and the Caribbean. Two studies evaluated World Paediatric Project missions to St Vincent and the Grenadines^44,45^; Dolan *et al*.^44^ (2021) reported I$5068 per DALY averted for trips between 2002 and 2019, using a threshold of half the country’s GDP per capita, while Goldfarb *et al*.^45^ (2023) found I$13 330 per DALY averted for upper limb surgeries between 2016 and 2019, applying a higher threshold.

Elsewhere in Latin America, mission trips demonstrated significant cost-effectiveness^47,50–52,57,58^. For example, Davis *et al*.^47^ (2014) reported an ICER of I$1030 per DALY averted from paediatric neurosurgeries in Guatemala. In Nicaragua, Taylor *et al*.^51^ (2021) found all surgeries during 16 trips by Esperanca between 2006 and 2014 to be cost-effective. In Honduras, Tadisinia *et al*.^50^ (2014) estimated I$1713 per DALY averted for hand surgeries and Eblovi *et al*.^58^ (2019) highlighted a US$17.9 million economic benefit from rural surgeries.

NGO missions in other regions were equally impactful. Hamze *et al*.^55^ (2017) estimated a $292 million economic gain from 37 274 cleft surgeries across East Africa. Globally, William Novick Cardiac Alliance’s paediatric cardiac surgeries achieved an ICER of I$171 per DALY averted^46^. Missions prioritizing local personnel training, such as those by the Touching Hands Project, yielded returns exceeding $3.5 million^48^. Overall, international surgical missions remain cost-effective and economically beneficial, particularly when combined with capacity-building efforts^49^.

### International missions *versus* domestic investments

Hackenberg *et al*.^54^ (2015) compared cleft lip and palate treatment strategies in India: 17 mission trips by the UK’s Operation Smile (2006–2012) *versus* a dedicated local specialist centre. Both were cost-effective by WHO thresholds, averting 6 DALYs per operation, but the specialist centre was less than half as expensive.

**Fig. 2:**
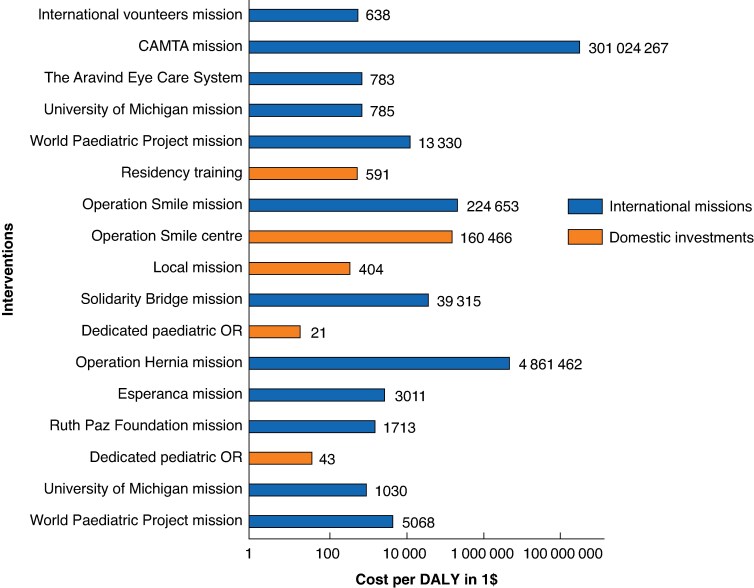
Cost per DALY of various international NGO missions and domestic interventions DALY, disability-adjusted life-year; NGO, non-governmental organization; CAMTA, Canadian Association of Medical Teams Abroad; OR, operating room; I$, international dollars.

Comparative analysis indicated that domestic interventions were significantly cheaper than international missions in terms of costs per benefit (QALYs gained or DALYs averted). Mean (standard deviation) and median (interquartile range) costs per benefit were I$27 051 (I$65 360) and I$498 (I$602), respectively, for domestic interventions, compared with I$515 500 (I$1 528 716) and I$5068 (I$31 618) for international missions. The difference was statistically significant (z = 2.412; *P* = 0.016). Details of the ACERs (costs per unit of benefit) are shown in *Fig. 2*.

### Quality assessment

The quality of studies varied, with inconsistent adherence to items in the Drummond checklist. All studies stated the research question and all but three reported the sources of effectiveness data. However, most lacked relevant clinical or demographic data for surgical patients and only three detailed the quantities of resources used separately from costs. Sensitivity analysis methods were clearly described in just over half of the studies. Full quality assessment details are provided in *Fig. S1*.

## Discussion

Evidence on the cost-effectiveness of interventions to promote access to surgery in LMICs published across 32 studies between January 2013 and January 2023 was synthesized and compared. Interventions included international missions and domestic initiatives such as investments in physical infrastructure, personnel training, national scale-up programmes, financial protection policies, and local surgical outreach activities. Most interventions were found to be cost-effective compared with no intervention or status quo. Within the limits of methodological heterogeneity, domestic surgical system investments commonly provided more value for money than international missions.

The findings of the present study align with existing evidence on the cost-effectiveness of surgery, which for decades has been considered prohibitively expensive for resource-poor populations^59,60^. Ifeanyichi *et al*.^16^ (2024) recently reviewed evidence regarding the cost-effectiveness of surgical procedures and found that common surgical procedures, most of which can be provided at district level (for example cataract surgeries, hernia repairs, and appendectomies), compared favourably with conventional public health interventions. This evidence provides the case for investments in appropriate local platforms for surgery. In addition to carrying substantial positive implications for population health, they may also impact on national economic development^2^.

International missions contribute significantly to surgical care provision across many LMICs, offering otherwise inaccessible surgical care to local populations. This study confirms that international missions are generally cost-effective, yet these findings must be interpreted in light of several underlying assumptions^11^. All included studies assumed that, without the missions, the beneficiaries would receive no surgical care. While this is plausible in many instances, significant surgical care initiatives are also provided by governments and private entities. In addition, studies almost exclusively discounted the risk of complications after surgery, which could be life-threatening and resource-intensive in terms of management, yet outside the costing frame of the initial mission^11,17^. These factors potentially bias the results in favour of missions, with evidence showing that missions become less cost-effective when compared with alternative domestic service delivery approaches^61^.

The findings of the present study add to the consensus that LMICs should be supported to develop local capacity and to prioritize investments in domestic systems capable of delivering safe, timely, and affordable surgical care, as they generally present a high return on investment^11,54^. Besides logistical implications (for example international flights, accommodation, etc.), which often increase the costs of the missions^45,52,53^, local investments typically benefit from economies of scale and scope to reduce unit costs of services provided^26,28,29^. For example, Emmett *et al*.^26^ (2019) reported that the volume of operations performed was a key determinant of cost-effectiveness for national cochlear implantation programmes, which diminishes the effect of lower GDP that otherwise makes such programmes appear too costly for LMICs.

Studies have also revealed other challenges associated with temporary mission-led programmes, including a lack of sustainability, a limited sensitivity to local health priorities, and a potential distortionary impact on the wider health system due to withdrawal of qualified personnel to vertical programmes^11,17^. However, the contributions of surgical missions to delivering care in LMICs should not be discounted, particularly in several low-income settings where missions are essential for addressing local needs efficiently^11^. Yet, missions must be considered as time-limited provisions only, to bridge a pressing care gap, while more sustainable systems are developed. They must be designed to target communities where they yield the most health and economic benefits, include robust arrangements for follow-up care, and contribute meaningfully to the transfer of skills to in-country collaborators. Development of international best practice guidelines for surgical missions may help to ensure reduction of possible harmful effects and to set out a path for a sustainable impact on local healthcare systems and populations.

The present study provides a holistic assessment of the cost-effectiveness of missions and domestic initiatives employed for the scale up of surgery in LMICs, with a broad search strategy and broad screening criteria. A wide range of strategies for improving delivery and uptake of surgical care was identified, with insights into potential economic values of investments in various settings. However, studies published before 2013 were excluded to prioritize the most current evidence, especially considering that economic evaluation evidence is sensitive to contemporary economic and financial states of the study setting, potentially missing research in this period. The study interval also aligned with an increased scholarly attention after publication of the 2015 *Lancet* Commission on Global Surgery, with more than half of the included articles building upon the report’s recommendations for scale up of national and local surgical capacity^1^.

Study methodology was heterogeneous and the overall poor quality of the evidence precluded more detailed comparison and meta-analysis. However, computation of ACERs, conversion of all values to 2022 I$, and the use of appropriate measures of central tendencies and dispersion enabled the derivation of valuable policy-relevant insights.

The cost-effectiveness of investments in surgical production factors (such as physical infrastructure and workforce training) and supply-side initiatives like universal public financing reinforces the increasing focus on integrating surgical care into UHC benefit packages. These strategies offer sustainable solutions compared with international missions, which, while addressing short-term surgical needs, risk adverse health outcomes for beneficiaries without adequate follow-up care and distortionary impacts on local health systems. To maximize population health benefits, a system-wide approach covering the care continuum is essential, aligning with World Health Assembly Resolution 76.2, which advocates for integrating emergency, critical, and operative care services.

## Supplementary Material

znaf207_Supplementary_Data

## Data Availability

All data used in this study are available upon reasonable request. **Previous communication**
