# Supplementary material for: Economic value of international missions and domestic initiatives to strengthen surgical care in low- and middle-income countries: systematic review
Source: Br J Surg. 2025 Dec 10;112(Suppl 15):xv18–29. doi: 10.1093/bjs/znaf207 (PMC12690695; doi:10.1093/bjs/znaf207)
Supplement: znaf207_Supplementary_Data [file znaf207_supplementary_data.docx]

**Economic Value of International Missions and Domestic Initiatives to Strengthen Surgical Care in Low- and Middle-Income Countries: A Systematic Review**

Martilord Ifeanyichi^1,2^, Yannis Reissis^1^, Rebecca Hakim^2,3^, Maeve Bognini^1^, Meskerem Kebede^1^, Rachel Hargest^1,4^, Rocco Friebel^1,5^

^1^Global Surgery Policy Unit, LSE Health, London School of Economics and Political Science, London, United Kingdom.

^2^Global Anaesthesia, Surgery and Obstetric Collaboration, Newcastle, United Kingdom

^3^Department of Surgery, University Hospitals Bristol and Weston NHS Foundation Trust, Bristol, United Kingdom

^4^School of Medicine, University Hospital of Wales, Cardiff, CF14 4XN, United Kingdom

^5^Department of Health Policy, London School of Economics and Political Science, London, United Kingdom

**Corresponding Author** Dr Martilord Ifeanyichi, Global Surgery Policy Unit, LSE Health, London School of Economics and Political Science, Cowdray House, Houghton Street, London, WC2A 2AE, United Kingdom. **ORCID ID** https://orcid.org/0000-0003-0611-6795 **Twitter** @Martilord **Email** [M.I.Ifeanyichi@lse.ac.uk](mailto:M.I.Ifeanyichi@lse.ac.uk).

**Supplementary Materials – Index**

[Supplementary Tables and Figures 2](#_Toc210725996)

[Supplementary Tables S1: Search strategy 2](#_Toc210725997)

[Supplementary Table S1a: Medline search strategy (OVID) 2](#_Toc210725998)

[Supplementary Table S1b: Embase search strategy (OVID) 4](#_Toc210725999)

[Supplementary Table S1c: Global Health search strategy (OVID) 7](#_Toc210726000)

[Supplementary Table S1d: EconLit search strategy (EBSCO) 10](#_Toc210726001)

[Supplementary Table S2: Inclusion and exclusion criteria 14](#_Toc210726002)

[Supplementary Table S3: Overview of results from the included studies 16](#_Toc210726003)

[Supplementary Figure S1: Rates of adherence with Drummond’s checklist 27](#_Toc210726004)

[References 28](#_Toc210726005)

# **Supplementary Tables and Figures**

## **Supplementary Tables S1: Search strategy**

### **Supplementary Table S1a: Medline search strategy (OVID)**

| **#** | (Surgery) AND (Cost-effectiveness) AND (LMIC) |  |
| --- | --- | --- |
| 1 | exp Specialties, Surgical/ or exp Anaesthesia/ or exp Surgical Procedures, Operative/ or surgery.fs. |  |
| 2 | (Anaesthe* or anesthe* or surger* or surgical).ti,ab,kf,kw. |  |
| 3 | 1 or 2 |  |
| 4 | Economics/ or exp "costs and cost analysis"/ or exp economics, hospital/ or exp economics, medical/ or economics, nursing/ or economics, pharmaceutical/ or economics, dental/ or value of life/ or exp "fees and charges"/ or exp budgets/ |  |
| 5 | (economic* or price or prices or pricing or expenditure or expenditures or expense or expenses or financial or finance or finances or financed or budget* or pharmacoeconomic* or pharmaco-economic* or fee or fees).ti,ab,kf,kw. |  |
| 6 | Cost*.ti. |  |
| 7 | (cost* adj2 (effective* or utilit* or benefit* or minimi* or consequence or analy* or outcome* or estimat* or variable*)).ab. |  |
| 8 | (value adj3 (money or monetary)).ti,ab,kf,kw. |  |
| 9 | exp Decision Theory/ or Markov Chains/ or Monte Carlo Method/ or exp Models, Economic/ or *models, theoretical/ or *models, organizational/ |  |
| 10 | (Markov* or econom* model* or monte carlo).ti,ab,kf,kw. |  |
| 11 | (Decision* adj2 (tree* or analy* or model*)).ti,ab,kf,kw. |  |
| 12 | Or/4-11 |  |
| 13 | (afghanistan or albania or algeria or american samoa or angola or "antigua and barbuda" or antigua or barbuda or argentina or armenia or armenian or aruba or azerbaijan or bahrain or bangladesh or barbados or republic of belarus or belarus or byelarus or belorussia or byelorussian or belize or british honduras or benin or dahomey or bhutan or bolivia or "bosnia and herzegovina" or bosnia or herzegovina or botswana or bechuanaland or brazil or brasil or bulgaria or burkina faso or burkina fasso or upper volta or burundi or urundi or cabo verde or cape verde or cambodia or kampuchea or khmer republic or cameroon or cameron or cameroun or central african republic or ubangi shari or chad or chile or china or colombia or comoros or comoro islands or iles comores or mayotte or congo or zaire or costa rica or "cote d’ivoire" or "cote d’ ivoire" or cote divoire or cote d ivoire or ivory coast or croatia or cuba or cyprus or czech republic or czechoslovakia or djibouti or french somaliland or dominica or dominican republic or ecuador or egypt or united arab republic or el salvador or equatorial guinea or spanish guinea or eritrea or estonia or eswatini or swaziland or ethiopia or fiji or gabon or gabonese republic or gambia or "georgia (republic)" or georgian or ghana or gold coast or gibraltar or greece or grenada or guam or guatemala or guinea or guinea bissau or guyana or british guiana or haiti or hispaniola or honduras or hungary or india or indonesia or timor or iran or iraq or isle of man or jamaica or jordan or kazakhstan or kazakh or kenya or korea or kosovo or kyrgyzstan or kirghizia or kirgizstan or kyrgyz republic or kirghiz or laos or lao pdr or "lao people's democratic republic" or latvia or lebanon or lebanese republic or lesotho or basutoland or liberia or libya or libyan arab jamahiriya or lithuania or macau or macao or republic of north macedonia or macedonia or madagascar or malagasy republic or malawi or nyasaland or malaysia or malay federation or Malaya Federation or maldives or indian ocean islands or indian ocean or mali or malta or micronesia or federated states of micronesia or kiribati or marshall islands or nauru or northern mariana islands or palau or tuvalu or mauritania or mauritius or mexico or moldova or moldovian or mongolia or montenegro or morocco or ifni or mozambique or portuguese east africa or myanmar or burma or namibia or nepal or netherlands antilles or nicaragua or niger or nigeria or oman or muscat or pakistan or panama or papua new guinea or new guinea or paraguay or peru or philippines or philipines or phillipines or phillippines or poland or "polish people's republic" or portugal or portuguese republic or puerto rico or romania or russia or russian federation or ussr or soviet union or union of soviet socialist republics or rwanda or ruanda or samoa or pacific islands or polynesia or samoan islands or navigator island or navigator islands or "sao tome and principe" or saudi arabia or senegal or serbia or seychelles or sierra leone or slovakia or slovak republic or slovenia or melanesia or solomon island or solomon islands or norfolk island or norfolk islands or somalia or south africa or south sudan or sri lanka or ceylon or "saint kitts and nevis" or "st. kitts and nevis" or saint lucia or "st. lucia" or "saint vincent and the grenadines" or saint vincent or "st. vincent" or grenadines or sudan or suriname or surinam or dutch guiana or netherlands guiana or syria or syrian arab republic or tajikistan or tadjikistan or tadzhikistan or tadzhik or tanzania or tanganyika or thailand or siam or timor leste or east timor or togo or togolese republic or tonga or "trinidad and tobago" or trinidad or tobago or tunisia or turkey or turkmenistan or turkmen or uganda or ukraine or uruguay or uzbekistan or uzbek or vanuatu or new hebrides or venezuela or vietnam or viet nam or middle east or west bank or gaza or palestine or yemen or yugoslavia or zambia or zimbabwe or northern rhodesia or global south or africa south of the sahara or sub-saharan africa or subsaharan africa or africa, central or central africa or africa, northern or north africa or northern africa or magreb or maghrib or sahara or africa, southern or southern africa or africa, eastern or east africa or eastern africa or africa, western or west africa or western africa or west indies or indian ocean islands or caribbean or central america or latin america or "south and central america" or south america or asia, central or central asia or asia, northern or north asia or northern asia or asia, southeastern or southeastern asia or south eastern asia or southeast asia or south east asia or asia, western or western asia or europe, eastern or east europe or eastern europe or developing country or developing countries or developing nation? or developing population? or developing world or less developed countr* or less developed nation? or less developed population? or less developed world or lesser developed countr* or lesser developed nation? or lesser developed population? or lesser developed world or under developed countr* or under developed nation? or under developed population? or under developed world or underdeveloped countr* or underdeveloped nation? or underdeveloped population? or underdeveloped world or middle income countr* or middle income nation? or middle income population? or low income countr* or low income nation? or low income population? or lower income countr* or lower income nation? or lower income population? or underserved countr* or underserved nation? or underserved population? or underserved world or under served countr* or under served nation? or under served population? or under served world or deprived countr* or deprived nation? or deprived population? or deprived world or poor countr* or poor nation? or poor population? or poor world or poorer countr* or poorer nation? or poorer population? or poorer world or developing econom* or less developed econom* or lesser developed econom* or under developed econom* or underdeveloped econom* or middle income econom* or low income econom* or lower income econom* or low gdp or low gnp or low gross domestic or low gross national or lower gdp or lower gnp or lower gross domestic or lower gross national or lmic or lmics or third world or lami countr* or transitional countr* or emerging economies or emerging nation?).ti,ab,sh,kf,kw. |  |
| 14 | 3 and 12 and 13 |  |
| 15 | Letter/ or editorial/ or case report/ or comment/ |  |
| 16 | (Exp animals/ or exp animals, laboratory/ or exp animal experimentation/ or exp models, animal/ or exp rodentia/ or (rat or rats or mouse or mice).ti.) not humans/ |  |
| 17 | (comment* or letter or editorial).ti. |  |
| 18 | 15 or 16 or 17 |  |
| 19 | 14 not 18 |  |
| 20 | Limit 19 to yr="2013-Current" |  |

**£** Low and Middle Income Terms – based on Norwegian Cochrane Collaboration LMIC filters ([LMIC Filters | Cochrane Effective Practice and Organisation of Care](https://epoc.cochrane.org/lmic-filters)); Low and lower-middle income countries based on World Bank listing (<https://datahelpdesk.worldbank.org/knowledgebase/articles/906519-world-bank-country-and-lending-groups>

### **Supplementary Table S1b: Embase search strategy (OVID)**

| **#** | **Embase Query**  (Surgery) AND (Cost-effectiveness) AND (LMIC) |  |
| --- | --- | --- |
| 1 | exp surgery/ OR surgery.fs. OR exp obstetric operation/ OR exp anaesthesia/ |  |
| 2 | (Anaesthe* or anesthe* or surger* or surgical).ti,ab,kf,kw. |  |
| 3 | 1 or 2 |  |
| 4 | Economics/ or exp "health economics"/ or cost/ or budget/ or funding/ |  |
| 5 | (Economic* or price or prices or pricing or expenditure or expenditures or expense or expenses or financial or finance or finances or financed or budget* or pharmacoeconomic* or pharmaco-economic* or fee or fees).ti,ab,kf,kw. |  |
| 6 | Cost*.ti. |  |
| 7 | (cost* adj2 (effective* or utilit* or benefit* or minimi* or consequence* or analy* or outcome* or estimat* or variable*)).ab. |  |
| 8 | (value adj3 (money or monetary)).ti,ab,kf,kw. |  |
| 9 | (Statistical model/ and exp economic aspect/) or stochastic model/ or *theoretical model/ or *nonbiological model/ or decision theory/ or decision tree/ or monte carlo method/ or probability/ |  |
| 10 | (Markov* or econom* model* or monte carlo).ti,ab,kf,kw. |  |
| 11 | (Decision* adj2 (tree* or analy* or model*)).ti,ab,kf,kw. |  |
| 12 | Or/4-11 |  |
| 13 | (afghanistan OR albania OR algeria OR american samoa OR angola OR "antigua and barbuda" OR antigua OR barbuda OR argentina OR armenia OR armenian OR aruba OR azerbaijan OR bahrain OR bangladesh OR barbados OR republic of belarus OR belarus OR byelarus OR belorussia OR byelorussian OR belize OR british honduras OR benin OR dahomey OR bhutan OR bolivia OR "bosnia and herzegovina" OR bosnia OR herzegovina OR botswana OR bechuanaland OR brazil OR brasil OR bulgaria OR burkina faso OR burkina fasso OR upper volta OR burundi OR urundi OR cabo verde OR cape verde OR cambodia OR kampuchea OR khmer republic OR cameroon OR cameron OR cameroun OR central african republic OR ubangi shari OR chad OR chile OR china OR colombia OR comoros OR comoro islands OR iles comores OR mayotte OR democratic republic of the congo OR democratic republic congo OR congo OR zaire OR costa rica OR "cote d’ivoire" OR "cote d’ ivoire" OR cote divoire OR cote d ivoire OR ivory coast OR croatia OR cuba OR cyprus OR czech republic OR czechoslovakia OR djibouti OR french somaliland OR dominica OR dominican republic OR ecuador OR egypt OR united arab republic OR el salvador OR equatorial guinea OR spanish guinea OR eritrea OR estonia OR eswatini OR swaziland OR ethiopia OR fiji OR gabon OR gabonese republic OR gambia OR "georgia (republic)" OR georgian OR ghana OR gold coast OR gibraltar OR greece OR grenada OR guam OR guatemala OR guinea OR guinea bissau OR guyana OR british guiana OR haiti OR hispaniola OR honduras OR hungary OR india OR indonesia OR timor OR iran OR iraq OR isle of man OR jamaica OR jordan OR kazakhstan OR kazakh OR kenya OR "democratic people’s republic of korea" OR republic of korea OR north korea OR south korea OR korea OR kosovo OR kyrgyzstan OR kirghizia OR kirgizstan OR kyrgyz republic OR kirghiz OR laos OR lao pdr OR "lao people's democratic republic" OR latvia OR lebanon OR lebanese republic OR lesotho OR basutoland OR liberia OR libya OR libyan arab jamahiriya OR lithuania OR macau OR macao OR republic of north macedonia OR macedonia OR madagascar OR malagasy republic OR malawi OR nyasaland OR malaysia OR malay federation OR malaya federation OR maldives OR indian ocean islands OR indian ocean OR mali OR malta OR micronesia OR federated states of micronesia OR kiribati OR marshall islands OR nauru OR northern mariana islands OR palau OR tuvalu OR mauritania OR mauritius OR mexico OR moldova OR moldovian OR mongolia OR montenegro OR "montenegro (republic)" OR morocco OR ifni OR mozambique OR portuguese east africa OR myanmar OR burma OR namibia OR nepal OR netherlands antilles OR nicaragua OR niger OR nigeria OR oman OR muscat OR pakistan OR panama OR papua new guinea OR new guinea OR paraguay OR peru OR philippines OR philipines OR phillipines OR phillippines OR poland OR "polish people's republic" OR portugal OR portuguese republic OR puerto rico OR romania OR russia OR russian federation OR ussr OR soviet union OR union of soviet socialist republics OR rwanda OR ruanda OR samoa OR pacific islands OR polynesia OR samoan islands OR navigator island OR navigator islands OR "sao tome and principe" OR saudi arabia OR senegal OR serbia OR seychelles OR sierra leone OR slovakia OR slovak republic OR slovenia OR melanesia OR solomon island OR solomon islands OR norfolk island OR norfolk islands OR somalia OR south africa OR south sudan OR sri lanka OR ceylon OR "saint kitts and nevis" OR "st. kitts and nevis" OR saint lucia OR "st. lucia" OR "saint vincent and the grenadines" OR saint vincent OR "st. vincent" OR grenadines OR sudan OR suriname OR surinam OR dutch guiana OR netherlands guiana OR syria OR syrian arab republic OR tajikistan OR tadjikistan OR tadzhikistan OR tadzhik OR tanzania OR tanganyika OR thailand OR siam OR timor leste OR east timor OR togo OR togolese republic OR tonga OR "trinidad and tobago" OR trinidad OR tobago OR tunisia OR "turkey (republic)" OR turkey OR turkmenistan OR turkmen OR uganda OR ukraine OR uruguay OR uzbekistan OR uzbek OR vanuatu OR new hebrides OR venezuela OR vietnam OR viet nam OR middle east OR west bank OR gaza OR palestine OR yemen OR yugoslavia OR zambia OR zimbabwe OR northern rhodesia OR global south OR africa south of the sahara OR "sub saharan africa" OR subsaharan africa OR africa, central OR central africa OR africa, northern OR north africa OR northern africa OR magreb OR maghrib OR sahara OR africa, southern OR southern africa OR africa, eastern OR east africa OR eastern africa OR africa, western OR west africa OR western africa OR west indies OR indian ocean islands OR caribbean region OR caribbean islands OR caribbean OR central america OR latin america OR "south and central america" OR south america OR asia, central OR central asia OR asia, northern OR north asia OR northern asia OR asia, southeastern OR southeastern asia OR south eastern asia OR southeast asia OR south east asia OR asia, western OR western asia OR europe, eastern OR east europe OR eastern europe OR developing country OR developing countries OR developing nation? OR developing population? OR developing world OR less developed countr* OR less developed nation? OR less developed population? OR less developed world OR lesser developed countr* OR lesser developed nation? OR lesser developed population? OR lesser developed world OR under developed countr* OR under developed nation? OR under developed population? OR under developed world OR underdeveloped countr* OR underdeveloped nation? OR underdeveloped population? OR underdeveloped world OR middle income countr* OR middle income nation? OR middle income population? OR low income countr* OR low income nation? OR low income population? OR lower income countr* OR lower income nation? OR lower income population? OR underserved countr* OR underserved nation? OR underserved population? OR underserved world OR under served countr* OR under served nation? OR under served population? OR under served world OR deprived countr* OR deprived nation? OR deprived population? OR deprived world OR poor countr* OR poor nation? OR poor population? OR poor world OR poorer countr* OR poorer nation? OR poorer population? OR poorer world OR developing econom* OR less developed econom* OR lesser developed econom* OR under developed econom* OR underdeveloped econom* OR middle income econom* OR low income econom* OR lower income econom* OR low gdp OR low gnp OR low gross domestic OR low gross national OR lower gdp OR lower gnp OR lower gross domestic OR lower gross national OR lmic OR lmics OR third world OR lami countr* OR transitional countr* OR emerging economies OR emerging nation?).ti,ab,sh,kw,kf. |  |
| 14 | 3 and 12 and 13 |  |
| 15 | Letter/ or editorial/ or case report/ or (letter or editorial).pt. |  |
| 16 | (comment* or letter or editorial).ti. |  |
| 17 | (exp animal/ or nonhuman/) not exp human/ |  |
| 18 | 15 or 16 or 17 |  |
| 19 | 1. not 18 |  |
| 20 | Limit 19 to yr="2013-Current" |  |

### **Supplementary Table S1c: Global Health search strategy (OVID)**

| **#** | (Surgery) AND (Cost-effectiveness) AND (LMIC) |  |
| --- | --- | --- |
| 1 | exp surgery/ OR exp surgical operations/ OR exp anaesthesia/ |  |
| 2 | (Anaesthe* or anesthe* or surger* or surgical).tw. |  |
| 3 | 1 or 2 |  |
| 4 | Economics/ or exp economic analysis/ or costs/ or health care costs/ or economic evaluation/ or fees/ or budgets/ or funding/ |  |
| 5 | (economic* or price or prices or pricing or expenditure or expenditures or expense or expenses or financial or finance or finances or financed or budget* or pharmacoeconomic* or pharmaco-economic* or fee or fees).tw. |  |
| 6 | Cost*.ti. |  |
| 7 | (cost* adj2 (effective* or utilit* or benefit* or minimi* or consequence* or analy* or outcome* or estimat* or variable*)).ab. |  |
| 8 | (value adj3 (money or monetary)).tw. |  |
| 9 | Markov processes/ or Monte carlo method/ or stochastic processes/ or statistical methods/ or probabilistic models/ or decision analysis/ or probability/ or stochastic model/ |  |
| 10 | (Markov* or econom* model* or monte carlo).tw. |  |
| 11 | (Decision* adj2 (tree* or analy* or model*)).tw. |  |
| 12 | Or/4-11 |  |
| 13 | (afghanistan or albania or algeria or american samoa or angola or "antigua and barbuda" or antigua or barbuda or argentina or armenia or armenian or aruba or azerbaijan or bahrain or bangladesh or barbados or republic of belarus or belarus or byelarus or belorussia or byelorussian or belize or british honduras or benin or dahomey or bhutan or bolivia or "bosnia and herzegovina" or bosnia or herzegovina or botswana or bechuanaland or brazil or brasil or bulgaria or burkina faso or burkina fasso or upper volta or burundi or urundi or cabo verde or cape verde or cambodia or kampuchea or khmer republic or cameroon or cameron or cameroun or central african republic or ubangi shari or chad or chile or china or colombia or comoros or comoro islands or iles comores or mayotte or congo or zaire or costa rica or "cote d’ivoire" or "cote d’ ivoire" or cote divoire or cote d ivoire or ivory coast or croatia or cuba or cyprus or czech republic or czechoslovakia or djibouti or french somaliland or dominica or dominican republic or ecuador or egypt or united arab republic or el salvador or equatorial guinea or spanish guinea or eritrea or estonia or eswatini or swaziland or ethiopia or fiji or gabon or gabonese republic or gambia or "georgia (republic)" or georgian or ghana or gold coast or gibraltar or greece or grenada or guam or guatemala or guinea or guinea bissau or guyana or british guiana or haiti or hispaniola or honduras or hungary or india or indonesia or timor or iran or iraq or isle of man or jamaica or jordan or kazakhstan or kazakh or kenya or korea or kosovo or kyrgyzstan or kirghizia or kirgizstan or kyrgyz republic or kirghiz or laos or lao pdr or "lao people's democratic republic" or latvia or lebanon or lebanese republic or lesotho or basutoland or liberia or libya or libyan arab jamahiriya or lithuania or macau or macao or republic of north macedonia or macedonia or madagascar or malagasy republic or malawi or nyasaland or malaysia or malay federation or Malaya Federation or maldives or indian ocean islands or indian ocean or mali or malta or micronesia or federated states of micronesia or kiribati or marshall islands or nauru or northern mariana islands or palau or tuvalu or mauritania or mauritius or mexico or moldova or moldovian or mongolia or montenegro or morocco or ifni or mozambique or portuguese east africa or myanmar or burma or namibia or nepal or netherlands antilles or nicaragua or niger or nigeria or oman or muscat or pakistan or panama or papua new guinea or new guinea or paraguay or peru or philippines or philipines or phillipines or phillippines or poland or "polish people's republic" or portugal or portuguese republic or puerto rico or romania or russia or russian federation or ussr or soviet union or union of soviet socialist republics or rwanda or ruanda or samoa or pacific islands or polynesia or samoan islands or navigator island or navigator islands or "sao tome and principe" or saudi arabia or senegal or serbia or seychelles or sierra leone or slovakia or slovak republic or slovenia or melanesia or solomon island or solomon islands or norfolk island or norfolk islands or somalia or south africa or south sudan or sri lanka or ceylon or "saint kitts and nevis" or "st. kitts and nevis" or saint lucia or "st. lucia" or "saint vincent and the grenadines" or saint vincent or "st. vincent" or grenadines or sudan or suriname or surinam or dutch guiana or netherlands guiana or syria or syrian arab republic or tajikistan or tadjikistan or tadzhikistan or tadzhik or tanzania or tanganyika or thailand or siam or timor leste or east timor or togo or togolese republic or tonga or "trinidad and tobago" or trinidad or tobago or tunisia or turkey or turkmenistan or turkmen or uganda or ukraine or uruguay or uzbekistan or uzbek or vanuatu or new hebrides or venezuela or vietnam or viet nam or middle east or west bank or gaza or palestine or yemen or yugoslavia or zambia or zimbabwe or northern rhodesia or global south or africa south of sahara or sub-saharan africa or subsaharan africa or africa, central or central africa or africa, northern or north africa or northern africa or magreb or maghrib or sahara or africa, southern or southern africa or africa, eastern or east africa or eastern africa or africa, western or west africa or western africa or west indies or indian ocean islands or caribbean or central america or latin america or "south and central america" or south america or asia, central or central asia or asia, northern or north asia or northern asia or asia, southeastern or southeastern asia or south eastern asia or southeast asia or south east asia or asia, western or western asia or europe, eastern or east europe or eastern europe or developing country or developing countries or developing nation? or developing population? or developing world or less developed countr* or less developed nation? or less developed population? or less developed world or lesser developed countr* or lesser developed nation? or lesser developed population? or lesser developed world or under developed countr* or under developed nation? or under developed population? or under developed world or underdeveloped countr* or underdeveloped nation? or underdeveloped population? or underdeveloped world or middle income countr* or middle income nation? or middle income population? or low income countr* or low income nation? or low income population? or lower income countr* or lower income nation? or lower income population? or underserved countr* or underserved nation? or underserved population? or underserved world or under served countr* or under served nation? or under served population? or under served world or deprived countr* or deprived nation? or deprived population? or deprived world or poor countr* or poor nation? or poor population? or poor world or poorer countr* or poorer nation? or poorer population? or poorer world or developing econom* or less developed econom* or lesser developed econom* or under developed econom* or underdeveloped econom* or middle income econom* or low income econom* or lower income econom* or low gdp or low gnp or low gross domestic or low gross national or lower gdp or lower gnp or lower gross domestic or lower gross national or lmic or lmics or third world or lami countr* or transitional countr* or emerging economies or emerging nation? Or Least developed countries or low human development index countries or low income countries or lower-middle income countries or medium human development index countries).ti,ab,sh. |  |
| 14 | 3 and 12 and 13 |  |
| 15 | "Letters (correspondence)"/ or editorial/ or case reports/ |  |
| 16 | (comment* or letter or editorial).ti. |  |
| 17 | 15 or 16 |  |
| 18 | 14 not 17 |  |
| 19 | Limit 18 to yr="2013-Current" |  |

### **Supplementary Table S1d: EconLit search strategy (EBSCO)**

| **#** | (Surgery) AND (Cost-effectiveness) AND (LMIC) |  |
| --- | --- | --- |
| 1 | TI(Anaesthe* or anesthe* or surger* or surgical) OR AB(Anaesthe* or anesthe* or surger* or surgical) |  |
| 2 | TI(economic* or price or prices or pricing or expenditure or expenditures or expense or expenses or financial or finance or finances or financed or budget* or pharmacoeconomic* or pharmaco-economic* or fee*) OR AB(economic* or price or prices or pricing or expenditure or expenditures or expense or expenses or financial or finance or finances or financed or budget* or pharmacoeconomic* or pharmaco-economic* or fee or fees) |  |
| 3 | TI(Cost*) |  |
| 4 | AB(cost* N2 (effective* or utilit* or benefit* or minimi* or consequence or analy* or outcome* or estimat* or variable*)) |  |
| 5 | TI(value N2 (money or monetary)) OR AB(value N2 (money or monetary)) |  |
| 6 | TI(Markov* or "econom* model*" or "monte carlo") or AB(Markov* or "econom* model*" or "monte carlo") |  |
| 7 | TI(Decision* N2 (tree* or analy* or model*)) OR AB(Decision* N2 (tree* or analy* or model*)) |  |
| 8 | S2 OR S3 OR S4 OR S5 OR S6 OR S7 |  |
| 9 | **TI**(afghanistan or albania or algeria or "american samoa" or angola or "antigua and barbuda" or antigua or barbuda or argentina or armenia or armenian or aruba or azerbaijan or bahrain or bangladesh or barbados or "republic of belarus" or belarus or byelarus or belorussia or byelorussian or belize or "British Honduras" or benin or dahomey or bhutan or bolivia or "bosnia and herzegovina" or bosnia or herzegovina or botswana or bechuanaland or brazil or brasil or bulgaria or "burkina faso" or "burkina fasso" or "upper volta" or burundi or urundi or "cabo verde" or "cape verde" or cambodia or kampuchea or "khmer republic" or cameroon or cameron or cameroun or "central african republic" or "ubangi shari" or chad or chile or china or colombia or comoros or "comoro islands" or "iles comores" or mayotte or congo or zaire or "costa rica" or "cote d’ivoire" or "cote d’ ivoire" or "cote divoire" or "cote d ivoire" or "ivory coast" or croatia or cuba or cyprus or "czech republic" or Czechoslovakia or djibouti or "french Somaliland" or dominica or "dominican republic" or ecuador or egypt or "united arab republic" or "el Salvador" or "equatorial guinea" or "spanish guinea" or eritrea or estonia or eswatini or swaziland or ethiopia or fiji or gabon or "gabonese republic" or gambia or "georgia (republic)" or georgian or ghana or "gold coast" or gibraltar or greece or grenada or guam or guatemala or guinea or "guinea Bissau" or guyana or "british Guiana" or haiti or hispaniola or honduras or hungary or india or indonesia or timor or iran or iraq or "isle of man" or jamaica or jordan or kazakhstan or kazakh or kenya or korea or kosovo or kyrgyzstan or kirghizia or kirgizstan or "kyrgyz republic" or kirghiz or laos or "lao pdr" or "lao people's democratic republic" or latvia or lebanon or "lebanese republic" or lesotho or basutoland or liberia or libya or "libyan arab Jamahiriya" or lithuania or macau or macao or "republic of north Macedonia" or macedonia or madagascar or "malagasy republic" or malawi or nyasaland or malaysia or "malay federation" or "Malaya Federation" or maldives or "indian ocean islands" or "indian ocean" or mali or malta or micronesia or "federated states of Micronesia" or kiribati or "marshall islands" or nauru or "northern mariana islands" or palau or tuvalu or mauritania or mauritius or mexico or moldova or moldovian or mongolia or montenegro or morocco or ifni or mozambique or "portuguese east Africa" or myanmar or burma or namibia or nepal or "netherlands Antilles" or nicaragua or niger or nigeria or oman or muscat or pakistan or panama or "papua new guinea" or "new guinea" or paraguay or peru or philippines or philipines or phillipines or phillippines or poland or "polish people's republic" or portugal or "portuguese republic" or "puerto rico" or romania or russia or "russian federation" or ussr or "soviet union" or "union of soviet socialist republics" or rwanda or ruanda or samoa or "pacific islands" or polynesia or "samoan islands" or "navigator island" or "navigator islands" or "sao tome and principe" or "saudi arabia" or senegal or serbia or seychelles or "sierra leone" or slovakia or "slovak republic" or slovenia or melanesia or "solomon island" or "solomon islands" or "norfolk island" or "norfolk islands" or somalia or "South Africa" or "south sudan" or "sri lanka" or ceylon or "saint kitts and nevis" or "st. kitts and nevis" or "saint lucia" or "st. lucia" or "saint vincent and the grenadines" or "saint Vincent" or "st. vincent" or grenadines or sudan or suriname or surinam or "dutch Guiana" or "netherlands Guiana" or syria or "syrian arab republic" or tajikistan or tadjikistan or tadzhikistan or tadzhik or tanzania or tanganyika or thailand or siam or "timor leste" or "east timor" or togo or "togolese republic" or tonga or "trinidad and tobago" or trinidad or tobago or tunisia or turkey or turkmenistan or turkmen or uganda or ukraine or uruguay or uzbekistan or uzbek or vanuatu or "new Hebrides" or venezuela or vietnam or "viet nam" or "middle east" or "west bank" or gaza or palestine or yemen or yugoslavia or zambia or zimbabwe or "northern Rhodesia" or "global south" or "africa south of the sahara" or "sub-saharan Africa" or "subsaharan Africa" or "africa, central" or "central Africa" or "africa, northern" or "north Africa" or "northern Africa" or magreb or maghrib or sahara or "africa, southern" or "southern Africa" or "africa, eastern" or "east Africa" or "eastern Africa" or "africa, western" or "west Africa" or "western Africa" or "west indies" or "indian ocean islands" or caribbean or "central America" or "latin America" or "south and central america" or "south America" or "asia, central" or "central asia" or "asia, northern" or "north asia" or "northern asia" or "asia, southeastern" or "southeastern asia" or "south eastern asia" or "southeast asia" or "south east asia" or "asia, western" or "western asia" or "europe, eastern" or "east Europe" or "eastern Europe" or "developing country" or "developing countries" or "developing nation#" or "developing population#" or "developing world" or "less developed countr*" or "less developed nation#" or "less developed population#" or "less developed world" or "lesser developed countr*" or "lesser developed nation#" or "lesser developed population#" or "lesser developed world" or "under developed countr*" or "under developed nation#" or "under developed population#" or "under developed world" or "underdeveloped countr*" or "underdeveloped nation#" or "underdeveloped population#" or "underdeveloped world" or "middle income countr*" or "middle income nation#" or "middle income population#" or "low income countr*" or "low income nation#" or "low income population#" or "lower income countr*" or "lower income nation#" or "lower income population#" or "underserved countr*" or "underserved nation#" or "underserved population#" or "underserved world" or "under served countr*" or "under served nation#" or "under served population#" or "under served world" or "deprived countr*" or "deprived nation#" or "deprived population#" or "deprived world" or "poor countr*" or "poor nation#" or "poor population#" or "poor world" or "poorer countr*" or "poorer nation#" or "poorer population#" or "poorer world" or "developing econom*" or "less developed econom*" or "lesser developed econom*" or "under developed econom*" or "underdeveloped econom*" or "middle income econom*" or "low income econom*" or "lower income econom*" or "low gdp" or "low gnp" or "low gross domestic" or "low gross national" or "lower gdp" or "lower gnp" or "lower gross domestic" or "lower gross national" or lmic or lmics or "third world" or "lami countr*" or "transitional countr*" or "emerging economies" or "emerging nation#") OR **AB**(afghanistan or albania or algeria or "american samoa" or angola or "antigua and barbuda" or antigua or barbuda or argentina or armenia or armenian or aruba or azerbaijan or bahrain or bangladesh or barbados or republic of belarus or belarus or byelarus or belorussia or byelorussian or belize or "british Honduras" or benin or dahomey or bhutan or bolivia or "bosnia and herzegovina" or bosnia or herzegovina or botswana or bechuanaland or brazil or brasil or bulgaria or "burkina faso" or "burkina fasso" or "upper volta" or burundi or urundi or "cabo verde" or "cape verde" or cambodia or kampuchea or "khmer republic" or cameroon or cameron or cameroun or "central african republic" or "ubangi shari" or chad or chile or china or colombia or comoros or "comoro islands" or "iles comores" or mayotte or congo or zaire or "costa rica" or "cote d’ivoire" or "cote d’ ivoire" or "cote divoire" or "cote d ivoire" or "ivory coast" or croatia or cuba or cyprus or "czech republic" or Czechoslovakia or djibouti or "french Somaliland" or dominica or "dominican republic" or ecuador or egypt or "united arab republic" or "el Salvador" or "equatorial guinea" or "spanish guinea" or eritrea or estonia or eswatini or swaziland or ethiopia or fiji or gabon or "gabonese republic" or gambia or "georgia (republic)" or georgian or ghana or "gold coast" or gibraltar or greece or grenada or guam or guatemala or guinea or "guinea Bissau" or guyana or "british Guiana" or haiti or hispaniola or honduras or hungary or india or indonesia or timor or iran or iraq or "isle of man" or jamaica or jordan or kazakhstan or kazakh or kenya or korea or kosovo or kyrgyzstan or kirghizia or kirgizstan or "kyrgyz republic" or kirghiz or laos or "lao pdr" or "lao people's democratic republic" or latvia or lebanon or "lebanese republic" or lesotho or basutoland or liberia or libya or "libyan arab Jamahiriya" or lithuania or macau or macao or "republic of north Macedonia" or macedonia or madagascar or "malagasy republic" or malawi or nyasaland or malaysia or "malay federation" or "Malaya Federation" or maldives or "indian ocean islands" or "indian ocean" or mali or malta or micronesia or "federated states of Micronesia" or kiribati or "marshall islands" or nauru or "northern mariana islands" or palau or tuvalu or mauritania or mauritius or mexico or moldova or moldovian or mongolia or montenegro or morocco or ifni or mozambique or "portuguese east Africa" or myanmar or burma or namibia or nepal or "netherlands Antilles" or nicaragua or niger or nigeria or oman or muscat or pakistan or panama or "papua new guinea" or "new guinea" or paraguay or peru or philippines or philipines or phillipines or phillippines or poland or "polish people's republic" or portugal or "portuguese republic" or "puerto rico" or romania or russia or "russian federation" or ussr or "soviet union" or "union of soviet socialist republics" or rwanda or ruanda or samoa or "pacific islands" or polynesia or "samoan islands" or "navigator island" or "navigator islands" or "sao tome and principe" or "saudi arabia" or senegal or serbia or seychelles or "sierra leone" or slovakia or "slovak republic" or slovenia or melanesia or "solomon island" or "solomon islands" or "norfolk island" or "norfolk islands" or somalia or "South Africa" or "south sudan" or "sri lanka" or ceylon or "saint kitts and nevis" or "st. kitts and nevis" or "saint lucia" or "st. lucia" or "saint vincent and the grenadines" or "saint Vincent" or "st. vincent" or grenadines or sudan or suriname or surinam or "dutch Guiana" or "netherlands Guiana" or syria or "syrian arab republic" or tajikistan or tadjikistan or tadzhikistan or tadzhik or tanzania or tanganyika or thailand or siam or "timor leste" or "east timor" or togo or "togolese republic" or tonga or "trinidad and tobago" or trinidad or tobago or tunisia or turkey or turkmenistan or turkmen or uganda or ukraine or uruguay or uzbekistan or uzbek or vanuatu or "new Hebrides" or venezuela or vietnam or "viet nam" or "middle east" or "west bank" or gaza or palestine or yemen or yugoslavia or zambia or zimbabwe or "northern Rhodesia" or "global south" or "africa south of the sahara" or "sub-saharan Africa" or "subsaharan Africa" or "africa, central" or "central Africa" or "africa, northern" or "north Africa" or "northern Africa" or magreb or maghrib or sahara or "africa, southern" or "southern Africa" or "africa, eastern" or "east Africa" or "eastern Africa" or "africa, western" or "west Africa" or "western Africa" or "west indies" or "indian ocean islands" or caribbean or "central America" or "latin America" or "south and central america" or "south America" or "asia, central" or "central asia" or "asia, northern" or "north asia" or "northern asia" or "asia, southeastern" or "southeastern asia" or "south eastern asia" or "southeast asia" or "south east asia" or "asia, western" or "western asia" or "europe, eastern" or "east Europe" or "eastern Europe" or "developing country" or "developing countries" or "developing nation#" or "developing population#" or "developing world" or "less developed countr*" or "less developed nation#" or "less developed population#" or "less developed world" or "lesser developed countr*" or "lesser developed nation#" or "lesser developed population#" or "lesser developed world" or "under developed countr*" or "under developed nation#" or "under developed population#" or "under developed world" or "underdeveloped countr*" or "underdeveloped nation#" or "underdeveloped population#" or "underdeveloped world" or "middle income countr*" or "middle income nation#" or "middle income population#" or "low income countr*" or "low income nation#" or "low income population#" or "lower income countr*" or "lower income nation#" or "lower income population#" or "underserved countr*" or "underserved nation#" or "underserved population#" or "underserved world" or "under served countr*" or "under served nation#" or "under served population#" or "under served world" or "deprived countr*" or "deprived nation#" or "deprived population#" or "deprived world" or "poor countr*" or "poor nation#" or "poor population#" or "poor world" or "poorer countr*" or "poorer nation#" or "poorer population#" or "poorer world" or "developing econom*" or "less developed econom*" or "lesser developed econom*" or "under developed econom*" or "underdeveloped econom*" or "middle income econom*" or "low income econom*" or "lower income econom*" or "low gdp" or "low gnp" or "low gross domestic" or "low gross national" or "lower gdp" or "lower gnp" or "lower gross domestic" or "lower gross national" or lmic or lmics or "third world" or "lami countr*" or "transitional countr*" or "emerging economies" or "emerging nation#") |  |
| 10 | S1 and S8 and S9 |  |
| 11 | TI(comment* OR letter or editorial OR "case report") |  |
| 12 | S10 NOT S11 |  |

## **Supplementary Table S2: Inclusion and exclusion criteria**

| Category | Inclusion | Exclusion |
| --- | --- | --- |
| Population | - Surgery - Anaesthesia - Obstetric - Surgical gynaecological | - Non-surgery - Medical obstetric - Medical gynaecological |
| Intervention | - Domestic policies, initiatives, investments, and programmes towards scaling up access to surgery. - International missions | - Specific surgical conditions |
| Comparator | - Policies, initiatives, investments, and programmes towards scaling up access to surgery. - International missions | - Specific surgical conditions |
| Outcomes | - Cost-effectiveness - Incremental Cost Effectiveness Ratios - Average Cost Effectiveness Ratios | - Clinical effectiveness - Costs (alone) |
| Setting | - Hospitals - Health centres - Low- and middle-income countries | - High-income countries |
| Study design | - Cost-effectiveness analyses - Cost-benefit analyses - Cost-utility analyses | - Cost minimisation analyses - Costing studies - Cost-consequence analysis - Cost saving analysis - Cost comparison analysis - Animal studies - Opinions/editorials/commentaries |
| Language | - All languages |  |
| Time limit | - January 2013 to January 2023 | - Studies published prior to January 2013 |

## **Supplementary Table S3: Overview of results from the included studies**

| S/N | Author, year  Country | Intervention | Comparator | Horizon | Cost (Int) | Cost (Comp) | Effects (Int) | Effects (Comp) | Unit of costs | Unit of effects |
| --- | --- | --- | --- | --- | --- | --- | --- | --- | --- | --- |
| **Domestic interventions** | | | | | | | | | | |
| 1 | Shrime, 2016(1) | Six scenarios to cover nine surgeries in rural Ethiopia   1. Universal public finance 2. Universal public finance + vouchers 3. Task sharing 4. Universal public finance + task sharing 5. Universal public finance + task sharing + vouchers 6. Task sharing + vouchers | Status quo (majority of surgeries are performed in urban areas) | Single event analytic horizon | 1. $944,000 2. $5,515,000 3. $400,000 4. $2,353,000 5. $9,705,000 6. $3,200,000 | Not reported; on average patients pay 34% of medical costs out of pocket prior to the intervention | Cases of poverty averted; number of deaths averted (per one million people, per $100,000 spent)   1. 38; 2 2. 48; 1 3. +145 cases of poverty; 64 4. +10 cases of poverty; 12 5. 27; 3 6. +12 cases of poverty; 9 | Not reported | I$ | Cases of poverty/CHE averted and number of deaths averted per $100,000 spent; incremental government costs |
| 2 | Verguet, 2015(2) | Universal public financing for caesarean section in rural Ethiopia | Universal public financing for eight primary health care interventions  (Rotavirus vaccine;  Pneumococcal vaccine; Measles vaccine; Diarrhoea treatment; Pneumonia treatment; Malaria treatment; TB treatment; Hypertension treatment) | Not reported | $420,000 | Rotavirus vaccine $800,000;  Pneumococcal vaccine $1,200,000;  Measles vaccine $260,000;  Diarrhoea treatment $50,000,000;  Pneumonia treatment $31,000,000;  Malaria treatment $670,000;  TB treatment $6,900,000;  Hypertension treatment $1,300,000 | Cases of poverty averted; number of deaths averted per $100,000 spent  98; 141 | Cases of poverty averted; number of deaths averted per $100,000 spent  Rotavirus vaccine 270; 510  Pneumococcal vaccine 170; 1,700  Measles vaccine 14; 890  Diarrhoea treatment 40,000; 3,600  Pneumonia treatment 23,000; 4,100  Malaria treatment 460; 410  TB treatment 6,700; 2,600  Hypertension treatment 1,100; 140 | USD | Cases of poverty averted; number of deaths averted per $100,000 spent |
| 3 | Essue, 2020(3) | Two scenarios for cataract surgery   1. Remove medical out-of-pocket costs 2. Remove medical and non-medical out-of-pocket costs | Status quo (patients pay all out-of-pocket costs) | Not reported | 1. I$1,858,161 2. I$3,718,046 | Current cost of phacoemulsification surgery I$441  Current cost of small incision surgery I$170 | 252 DALYs averted | Not reported | I$ | DALYs averted |
| 4 | Emmett, 2019(4) | Cochlear implantation + mainstream education | Deaf education with sign language | 10 years | Total programme costs  Nepal $193,010,989  Bangladesh $666,905,768  Cambodia $72,906,301  Pakistan $1,797,365,197  India $7,161,257,582  Philippines $561,259,826  Indonesia $1,599,529,872  Sri Lanka $98,678,122 | Total programme costs  Nepal $91,943,514  Bangladesh $553,442,329  Cambodia $8,790,851  Pakistan $808,774,337  India $2,154,447,293  Philippines $61,973,623  Indonesia $530,019,630  Sri Lanka $20,505,572 | DALYs averted  Nepal 4.67  Bangladesh 4.72  Cambodia 4.58  Pakistan 4.63  India 4.61  Philippines 4.45  Indonesia 4.69  Sri Lanka 4.78 | DALYs averted  Nepal 3.95  Bangladesh 4  Cambodia 4.01  Pakistan 3.91  India 3.89  Philippines 3.9  Indonesia 3.92  Sri Lanka 4.05 | USD | DALYs averted |
| 5 | Emmett, 2015(5) | Cochlear implantation + mainstream education | Deaf education with sign language | 10 years | Total programme costs  South Africa $390,426,878  Nigeria $1,837,497,061  Kenya $371,341,635  Rwanda $80,992,482  Uganda $299,213,658  Malawi $117,620,136 | Total programme costs  South Africa $478,945,922  Nigeria $497,959,615  Kenya $83,884,540  Rwanda $7,326,654  Uganda $57,945,326  Malawi $8,860,548 | DALYs averted  South Africa 6.41  Nigeria 6.05  Kenya 6.45  Rwanda 6.67  Uganda 6.18  Malawi 6.32 | DALYs averted  South Africa 5.19  Nigeria 4.9  Kenya 5.26  Rwanda 5.47  Uganda 5  Malawi 5.13 | USD | DALYs averted |
| 6 | Emmett, 2016(6) | Cochlear implantation + mainstream education | Deaf education with sign language | 10 years | Total programme costs  Brazil $658,050,390  Cambodia $171,500,179  Ecuador $165,797,061  Guatemala $193,419,589  Paraguay $66,640,577  Trinidad and Tobago $11,377,679  Venezuela $322,157,750 | Total programme costs  Brazil $490,177,173  Cambodia $12,044,695  Ecuador $17,254,284  Guatemala $48,593,224  Paraguay $14,380,630  Trinidad and Tobago $9,995,183  Venezuela $68,764,616 | DALYs averted  Brazil 4.34  Cambodia 4.48  Ecuador 4.63  Guatemala 4.41  Paraguay 4.76  Trinidad and Tobago 4.43  Venezuela 4.02 | DALYs averted  Brazil 4.04  Cambodia 4.01  Ecuador 4.07  Guatemala 3.94  Paraguay 4.02  Trinidad and Tobago 3.96  Venezuela 4.02 | USD | DALYs averted |
| 7 | Bansi-Matharu, 2023(7) | Circumcision for 15-64 year-olds for five years | No further circumcision | 50 years | Mean annual total programme costs per 10m adults over time horizon ($m/year)  SOUTH AFRICA   \| Goals-ASM \|  \| \| --- \| --- \| \| 5 years \| $407 \| \| 20 years \| $255 \| \| 50 years \| $152 \| \| Optima HIV \|  \| \| 5 years \| $275 \| \| 20 years \| $232 \| \| 50 years \| $175 \| \| EMOD \|  \| \| 5 years \| $296 \| \| 20 years \| $250 \| \| 50 years \| $163 \| \| Thembisa \|  \| \| 5 years \| $352 \| \| 20 years \| $266 \| \| 50 years \| $148 \| \| MALAWI \|  \| \| Goals-ASM \|  \| \| 5 years \| $232 \| \| 20 years \| $165 \| \| 50 years \| $113 \| \| Optima HIV \|  \| \| 5 years \| $151 \| \| 20 years \| $119 \| \| 50 years \| $85 \| \| ZIMBABWE \|  \| \| Goals-ASM \|  \| \| 5 years \| $287 \| \| 20 years \| $200 \| \| 50 years \| $124 \| \| Optima HIV \|  \| \| 5 years \| $262 \| \| 20 years \| $225 \| \| 50 years \| $181 \| \| HIV Synthesis \|  \| \| 5 years \| $149 \| \| 20 years \| $114 \| \| 50 years \| $80 \| | Mean annual total programme costs per 10m adults over time horizon ($m/year)  SOUTH AFRICA  Goals-ASM   \| 5 years \| $398 \| \| --- \| --- \| \| 20 years \| $254 \| \| 50 years \| $154 \| \| Optima HIV \|  \| \| 5 years \| $268 \| \| 20 years \| $230 \| \| 50 years \| $176 \| \| EMOD \|  \| \| 5 years \| $286 \| \| 20 years \| $249 \| \| 50 years \| $165 \| \| Thembisa \|  \| \| 5 years \| $346 \| \| 20 years \| $265 \| \| 50 years \| $150 \| \| MALAWI \|  \| \| Goals-ASM \|  \| \| 5 years \| $199 \| \| 20 years \| $159 \| \| 50 years \| $113 \| \| Optima HIV \|  \| \| 5 years \| $140 \| \| 20 years \| $116 \| \| 50 years \| $85 \| \| ZIMBABWE \|  \| \| Goals-ASM \|  \| \| 5 years \| $255 \| \| 20 years \| $193 \| \| 50 years \| $125 \| \| Optima HIV \|  \| \| 5 years \| $250 \| \| 20 years \| $221 \| \| 50 years \| $180 \| \| HIV Synthesis \|  \| \| 5 years \| $142 \| \| 20 years \| $113 \| \| 50 years \| $80 \| | Difference in net DALYs averted (mean/year)   \| SOUTH AFRICA \|  \| \| --- \| --- \| \| Goals-ASM \|  \| \| 5 years \| 67056 additional \| \| 20 years \| 17160 additional \| \| 50 years \| 64020 averted \| \| Optima HIV \|  \| \| 5 years \| 63186 additional \| \| 20 years \| 7971 additional \| \| 50 years \| 11552 averted \| \| EMOD \|  \| \| 5 years \| 114075 additional \| \| 20 years \| 6370 additional \| \| 50 years \| 72379 averted \| \| Thembisa \|  \| \| 5 years \| 50600 additional \| \| 20 years \| 8283 averted \| \| 50 years \| 42956 averted \| \| MALAWI \|  \| \| Goals-ASM \|  \| \| 5 years \| 67039 additional \| \| 20 years \| 5394 additional \| \| 50 years \| 11045 averted \| \| Optima HIV \|  \| \| 5 years \| 25506 additional \| \| 20 years \| 4548 additional \| \| 50 years \| 2259 averted \| \| ZIMBABWE \|  \| \| Goals-ASM \|  \| \| 5 years \| 51615 additional \| \| 20 years \| 6517 additional \| \| 50 years \| 6084 averted \| \| Optima HIV \|  \| \| 5 years \| 19801 additional \| \| 20 years \| 4083 additional \| \| 50 years \| 210 additional \| \| HIV Synthesis \|  \| \| 5 years \| 12096 additional \| \| 20 years \| 1284 additional \| \| 50 years \| 1267 averted \| | | USD | DALYs averted |
| 8 | Haacker, 2016(8) | Voluntary male circumcision performed in 2013 | No intervention | 80 years | \| $104 for adults \| \| --- \| \| $52 for infants \| | Not reported | Reduction in HIV incidence (age at circumcision: impact)   \| 0 years: 0.236 \| \| --- \| \| 10 years: 0.232 \| \| 15 years: 0.218 \| \| 20 years: 0.227 \| \| 25 years: 0.159 \| \| 30 years: 0.079 \| \| 35 years: 0.04 \| \| 40 years: 0.022 \| \| 45 years: 0.014 \| \| 50 years: 0.009 \| \| 55 years: 0.005 \| | Not reported | USD | HIV infections averted |
| 9 | Holmes, 2021(9) | Targeting strategy to circumcise men in the Gauteng province in 2015 | Status quo (routine outreach strategies) | 13 months | Strategy costs $9,445  HIV treatment costs averted due to intervention:  $542,491 public model $378,073 private model | $15,780 | 57 HIV cases averted | Not reported | USD | HIV cases averted |
| 10 | Bayani, 2021(10) | Three scenarios for renal replacement therapy   1. Adequate haemodialysis (PhilHealth covers three sessions/ week and costs of immune suppression) 2. Peritoneal dialysis first for all eligible patients 3. Peritoneal dialysis first and pre-emptive transplant | Status quo  (94% on haemodialysis, 4% on peritoneal dialysis, 2% receive transplant; immune suppression not covered by PhilHealth) | 10 years | Total lifetime costs per patient for each policy scenario   1. Health care provider perspective 4,885,920PHP; Societal perspective 5,812,280PHP 2. Health care provider perspective 2,065,700PHP; Societal perspective 2,275,470PHP 3. Health care provider perspective 2,119,960PHP; Societal perspective 2,312,160PHP | Total lifetime costs from a health care provider perspective  521,990PHP | Not reported | Not reported | PHP | QALYs gained |
| 11 | Nunes, 2018(11) | Scenario whereby Cape Verde performs hip replacements for both fractures and arthritis | Status quo (hip fractures are treated in Cape Verde and arthritis in Portugal) | 6 years | 135 total hip replacements  €404,736.8 | €485,380.88 | Not reported | Not reported | Euros | Not reported |
| 12 | Le, 2016(12) | 292 cataract surgeries performed in the Avarind Eye Care System | No surgery | Not reported | $28,891 | Not reported | 181 QALYs gained | Not reported | USD | QALYs gained |
| 13 | Watkins, 2016(13) | 2 policy scenarios for rheumatic heart disease   1. Build a surgical centre 2. Refer for surgery abroad | Scale up primary & secondary prevention | 100 years | Cost per capita   1. $25,626.54 2. $3,711.69 | Cost per capita  Primary prevention $3.66  Secondary prevention  $2,226.59 | 218 DALYs averted by surgical care | DALYs averted  Primary prevention 501  Secondary prevention 1025 | USD | DALYs averted |
| 14 | Yap, 2021(14) | Build a dedicated paediatric theatre | No theatre | 1 year | $244,001 | $0 | 6,551 DALYs averted (17.5 per patient) | Not reported | USD | DALYs averted |
| 15 | Yap, 2018(15) | Build a dedicated paediatric theatre | No theatre | Not reported | $101,847.57 | $0 | 8,604 DALYs averted | - | USD | DALYs averted |
| 16 | Agwu, 2021(16) | 5 years of orthopaedic residency training | No surgical training | Lifetime | Lifetime cost of one trainee $448,600 | $0 | 5,570 DALYs averted by each trainee throughout their career | - | USD | DALYs averted |
| 17 | Gyedu, 2017(17) | All outreach trips by the AMOG group between 2011-2016 | No surgery | Not reported | $283,762 | $0 | 2,079 DALYs averted | - | USD | DALYs averted |
| **International Missions** | | | | | | | | | | |
| 18 | Dolan, 2021(18) | 914 paediatric surgeries performed by the World Paediatric Project between 2002-19 | No surgery | Study period 2002-19 | $15,250,189.97 | $0 | 5,815 DALYs averted overall  DALYs averted by specialty:   \| Ophthalmic 1817 \| \| --- \| \| Orthopaedic 544 \| \| Plastics 1075 \| \| General Surgery 1544 \| \| Urology 106 \| \| Neurosurgery 729 \| | - | USD | DALYs averted |
| 19 | Goldfarb, 2021(19) | Three mission trips by the World Paediatric Project for upper limb surgeries between 2016-2019 | No surgery | Not reported | Total cost for St Vincent and the Grenadines patients $310,890.96  Total cost for non-St Vincent and the Grenadines patients $292,924.59 | $0 | 94.3 DALYs averted | - | USD | DALYs averted |
| 20 | Cardarelli, 2018(20) | 470 paediatric cardiac surgeries performed by the William Novick Cardiac Alliance in 2015 | No surgery | 1 year | Total programme service costs $3,210,873  Estimated cost per surgery $6,831  Costs of re-operation $34,155 | $0 | 39.9 DALYs averted and 3.5 years of extra schooling per surviving patient | - | USD | DALYs averted; years of schooling |
| 21 | Davis, 2014(21) | 17 paediatric neurosurgical operations performed in 2014 by the University of Michigan mission trip | No surgery  Same procedures performed at the University of Michigan | 1 week | $53,152.19 | $0 for no surgery  $679,427 for equivalent procedures performed at the University of Michigan | 138.1 DALYs averted  DALYs averted by operation (mean):  10.4 for ventriculoperitoneal shunt;  4.3 for thoracic meningocele repair;  6.3 for lipomyelomeningocele repair;  10.1 myelomeningocele repair;  0.1 for dermoid cyst excision;  23.8 for endoscopic third ventriculostomy | - | USD | DALYs averted |
| 22 | Billig, 2020(22) | 15 hand surgery mission trips by The Touching Hands Project and ReSurge International between 2015-2018 | No surgery | Not reported | Mean cost per mission trip $24,869 | $0 | Mean 1.5 DALYs averted per trip  Mean net economic benefit per trip: $238,456 using human capital approach; $576,716 using value of a statistical life year approach | - | USD | DALYs averted  Net economic benefit |
| 23 | Qiu, 2019(23) | 14 hand surgery mission trips by The Touching Hands Project and ReSurge International between 2015-2018 | No surgery | Not reported | Trip 1 $42,816  Trip 2 $44,308  Trip 3 $10,606  Trip 4 $10,462  Trip 5 $8,166  Trip 6 $5,324  Trip 7 $5,536  Trip 8 $3,453  Trip 9 $13,078  Trip 10 $87,434  Trip 11 $14,000  Trip 12 $84,045  Trip 13 $16,723  Trip 14 $16,372 | $0 | DALYs averted per trip:  Trip 1 0.84  Trip 2 0.98  Trip 3 2.24  Trip 4 2.5  Trip 5 1.78  Trip 6 0.77  Trip 7 0.75  Trip 8 1.37  Trip 9 2.03  Trip 10 1.17  Trip 11 2.26  Trip 12 1.52  Trip 13 1.16  Trip 14 2.54 | - | USD | DALYs averted |
| 24 | Tadisina, 2014(24) | Hand surgery mission trip by the Ruth Paz Foundation in 2006 | No surgery | Not reported | $45,779 | $0 | 104.6 DALYs averted | - | USD | DALYs averted |
| 25 | Taylor, 2021(25) | 563 operations performed during 16 mission trips by Esperanca between 2006-2014 | No surgery | Lifetime | Total cost $3,342,646.90  Costs per speciality:  General Surgery $1,367,466.20;  Paediatric Surgery $420,811.72;  Gynaecological Surgery $751,376.55;  Orthopaedic Surgery $781,233.72 | $0 | DALYs averted per specialty:  General Surgery 525.56;  Paediatric Surgery 283.15;  Gynaecological Surgery 431.81;  Orthopaedic Surgery 125.71 | - | USD | DALYs averted |
| 26 | Shillcutt, 2013(26) | 102 inguinal hernia repairs performed by Operation Hernia during two trips to a rural hospital in 2010 | No surgery | Not reported | $2,457.48 | $0 | 639 DALYs averted | - | USD | DALYs averted |
| 27 | Ament 2014(27) | 16 spinal surgeries performed by Solidarity Bridge between 2008-2011 | No surgery | 2 years | $9,036 | $0 | Total incremental QALY gain of 0.771 | - | USD | QALYs gained |
| 28 | Hamze, 2017(28) | 37,274 cleft lip and palate repairs performed by Smile Train between 2006-2014 | No surgery | Not reported | $13,045,900 | $0 | 207,879 DALYs averted | - | USD | DALYs averted |
| 29 | Egle, 2014(29) | General and gynaecological surgeries performed by Midwest Medical Missions Michigan Chapter during two trips between 2010-2012 | No surgery  Equivalent procedures performed at host institution | Not reported | $144,292 for 2010 and 2012 | Equivalent procedures at host institution:  $690,252 for 2010 and 2012 | 473 DALYs averted | - | USD | DALYs averted |
| 30 | Schlegelmilch, 2017(30) | 157 total hip replacements by CAMTA between 2007-2011 | No surgery | 1 year | Cost for all operations $1,107,996  Unilateral THR $6,042.45  Bilateral THR $7,229.13  Staged THR $12,786.08 | $0 | Lifetime QALYs gained:  Unilateral THR 1.46  Bilateral THR 2.51  Staged THR 2.93  Health utility by 15D scores (increase at one year):  Unilateral THR 10.3%  Bilateral THR 12.2%  Staged THR 14.1% | - | CAD | QALYs gained |
| 31 | Eblovi, 2019(31) | Mixture of general surgery, urology, orthopaedics, ENT, ophthalmology and gynaecology surgeries performed during trips by various HICs volunteers to a rural ambulatory centre in 2017 | No surgery | 1 year | $2,650,458 | $0 | 4,153.81 DALYs averted | - | USD | DALYs averted |
| **Comparison between mission trips and domestic interventions** | | | | | | | | | | |
| 32 | Hackenberg, 2015(32) | Two strategies for cleft lip & palate repair by Operation Smile between 2006-2012   1. 17 mission trips 2. Comprehensive care centre (2010-2012) | No surgery | Not reported | 1. I$5,218,159 2. I$3,148,365 | $0 | 1. 21,006 DALYs averted (average 6/patient) 2. 16,569 DALYs averted (average 6/patient) | - | I$ | DALYs averted |

Abbreviations: CHE = catastrophic health expenditure; I$ = International dollars; USD = US dollars; DALYs = Disability Adjusted Life Years; PHP = Philippine Pesos; QALYs = Quality Adjusted Life Years; THR = Total hip replacement; CAD = Canadian Dollars; HICs = high income countries

## **Supplementary Figure S1: Rates of adherence with Drummond’s checklist**

**
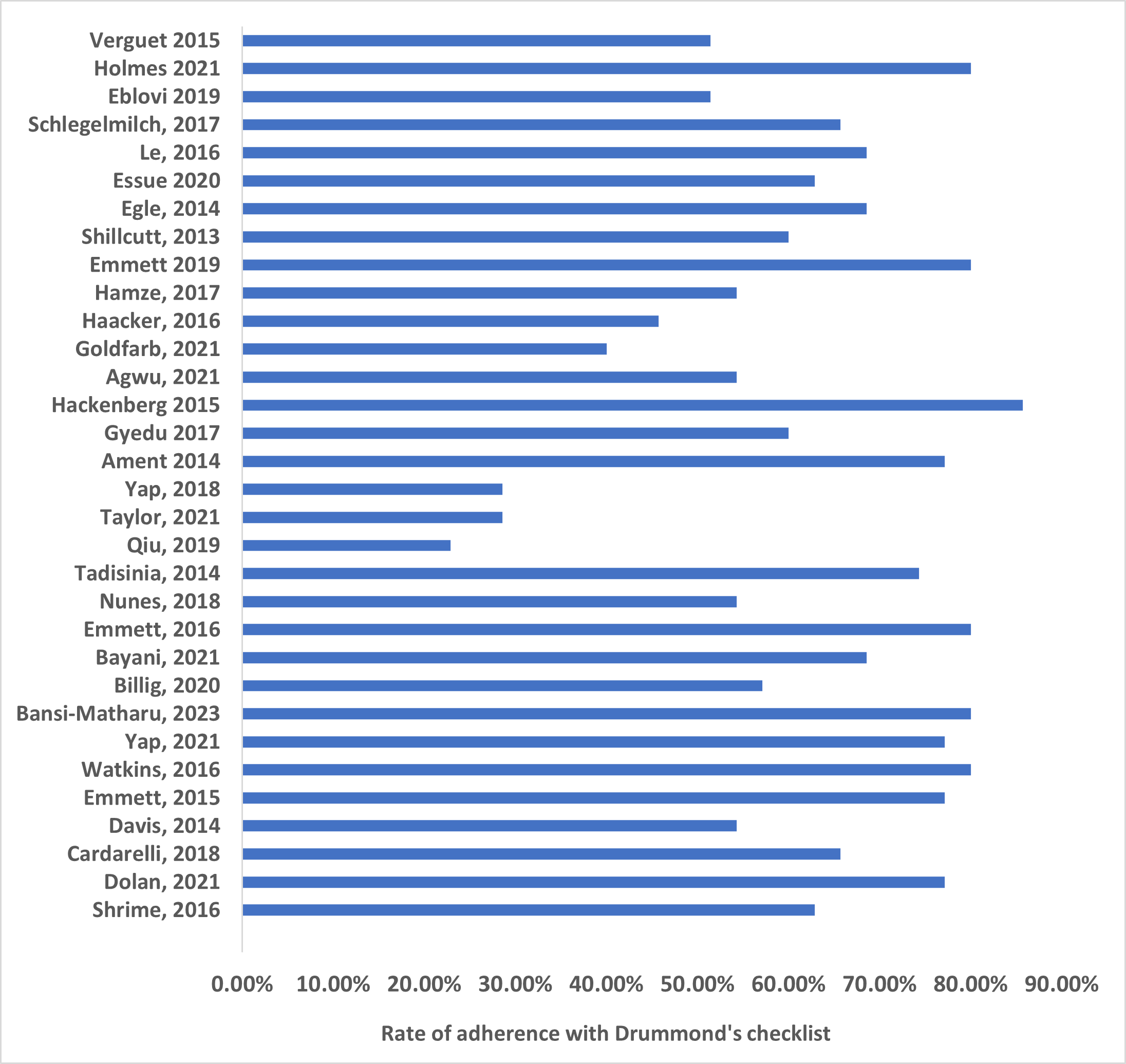
**

# **References**

1. Shrime MG, Verguet S, Johansson KA, Desalegn D, Jamison DT, Kruk ME. Task-Sharing or Public Finance for the Expansion of Surgical Access in Rural Ethiopia: An Extended Cost-Effectiveness Analysis. Health Policy and Planning. 2016 Jul;31(6):706–16.

2. Verguet S, Olson ZD, Babigumira JB, Desalegn D, Johansson KA, Kruk ME, et al. Health gains and financial risk protection afforded by public financing of selected interventions in Ethiopia: an extended cost-effectiveness analysis. The Lancet Global Health. 2015;3(5):e288–96.

3. Essue BM, Jan S, Phuc HT, Dodson S, Armstrong K, Laba TL. Who Benefits Most from Extending Financial Protection for Cataract Surgery in Vietnam? An Extended Cost-Effectiveness Analysis of Small Incision Surgery. Health Policy and Planning. 2020 May;35(4):399–407.

4. Emmett SD, Sudoko CK, Tucci DL, Gong W, Saunders JE, Global HEAR (Hearing Loss Evaluation A, et al. Expanding access: cost-effectiveness of cochlear implantation and deaf education in Asia. Otolaryngology–Head and Neck Surgery. 2019;161(4):672–82.

5. Emmett SD, Tucci DL, Smith M, Macharia IM, Ndegwa SN, Nakku D, et al. GDP matters: cost effectiveness of cochlear implantation and deaf education in Sub-Saharan Africa. Otology & Neurotology. 2015;36(8):1357–65.

6. Emmett SD, Tucci DL, Bento RF, Garcia JM, Juman S, Chiossone-Kerdel JA, et al. Moving beyond GDP: cost effectiveness of cochlear implantation and deaf education in Latin America. Otology & Neurotology. 2016;37(8):1040–8.

7. Bansi-Matharu L, Mudimu E, Martin-Hughes R, Hamilton M, Johnson L, Brink D ten, et al. Cost-effectiveness of voluntary medical male circumcision for HIV prevention across sub-Saharan Africa: results from five independent models. The Lancet Global Health. 2023 Feb 1;11(2):e244–55.

8. Haacker M, Fraser-Hurt N, Gorgens M. Effectiveness of and Financial Returns to Voluntary Medical Male Circumcision for HIV Prevention in South Africa: An Incremental Cost-Effectiveness Analysis. PLOS Medicine. 2016 May 3;13(5):e1002012.

9. Holmes M, Mukora R, Mudzengi D, Charalambous S, Chetty-Makkan CM, Kisbey-Green H, et al. An economic evaluation of an intervention to increase demand for medical male circumcision among men aged 25–49 years in South Africa. BMC Health Serv Res. 2021 Oct 15;21(1):1097.

10. Bayani DBS, Almirol BJQ, Uy GDC, Taneo MJS, Danguilan RS, Arakama MHI, et al. Filtering for the best policy: An economic evaluation of policy options for kidney replacement coverage in the Philippines. Nephrology (Carlton, Vic) [Internet]. 2020; Available from: http://ovidsp.ovid.com/ovidweb.cgi?T=JS&PAGE=reference&D=emexb&NEWS=N&AN=633463140

11. Nunes AM, Canhão H, Rodrigues TL. Enabling Cape Verde to Perform Total Hip Replacement: Cost-Benefit Study. Acta Médica Portuguesa. 2018 Dec 28;31(12):738–41.

12. Le HG, Ehrlich JR, Venkatesh R, Srinivasan A, Kolli A, Haripriya A, et al. A Sustainable Model For Delivering High-Quality, Efficient Cataract Surgery In Southern India. Health Affairs. 2016 Oct;35(10):1783–90.

13. Watkins D, Lubinga SJ, Mayosi B, Babigumira JB. A Cost-Effectiveness Tool to Guide the Prioritization of Interventions for Rheumatic Fever and Rheumatic Heart Disease Control in African Nations. PLOS Neglected Tropical Diseases. 2016 Aug 11;10(8):e0004860.

14. Yap A, Cheung M, Muzira A, Healy J, Kakembo N, Kisa P, et al. Best Buy in Public Health or Luxury Expense? Ann Surg. 2021 Feb 1;273(2):379–86.

15. Yap A, Muzira A, Cheung M, Healy J, Kakembo N, Kisa P, et al. A Cost-Effectiveness Analysis of a Pediatric Operating Room in Uganda. Surgery. 2018 Nov 1;164(5):953–9.

16. Agwu C, Purcell LN, Gallaher J, Young S, Banza L, Mansfield AJ, et al. Cost-Effectiveness analysis of the surgical management of fractures in Malawi: An economic evaluation of a high and low-income country surgical collaboration. Injury. 2021;52(4):767–73.

17. Gyedu A, Gaskill C, Boakye G, Abantanga F. Cost-effectiveness of a locally organized surgical outreach mission: making a case for strengthening local non-governmental organizations. World journal of surgery. 2017;41:3074–82.

18. Dolan CB, Agyemang SA, Clare B, Coleman C, Richter B, Robertson E, et al. Cost-effectiveness of paediatric surgery: an economic evaluation of World Paediatric Project surgical interventions in St. Vincent and the Grenadines (2002–2019). BMJ Open. 2021 Dec 25;11(12):e050286.

19. Goldfarb JH, Manteiga A, Wall LB. Cost-Effectiveness of Pediatric Hand International Medical Missions. The Journal of Hand Surgery. 2023 Mar 1;48(3):310.e1-310.e11.

20. Cardarelli M. Cost-effectiveness of Humanitarian Pediatric Cardiac Surgery Programs in Low- and Middle-Income Countries | Cardiology | JAMA Network Open | JAMA Network [Internet]. [cited 2024 Feb 19]. Available from: https://jamanetwork.com/journals/jamanetworkopen/article-abstract/2714503

21. Davis MC, Than KD, Garton HJ. Cost Effectiveness of a Short-Term Pediatric Neurosurgical Brigade to Guatemala. World Neurosurgery. 2014 Dec 1;82(6):974–9.

22. Billig JI, Nasser JS, Sue GR, Chang J, Chung KC. Economic Benefit of Hand Surgical Efforts in Low- and Middle-Income Countries: A Cost-Benefit Analysis. Plastic and reconstructive surgery [Internet]. 2019; Available from: http://ovidsp.ovid.com/ovidweb.cgi?T=JS&PAGE=reference&D=emexb&NEWS=N&AN=630273957

23. Qiu X, Nasser JS, Sue GR, Chang J, Chung KC. Cost-Effectiveness Analysis of Humanitarian Hand Surgery Trips According to WHO-CHOICE Thresholds. The Journal of Hand Surgery. 2019 Feb 1;44(2):93–103.

24. Tadisina KK, Chopra K, Tangredi J, Thomson JG, Singh DP. Helping Hands: A Cost-Effectiveness Study of a Humanitarian Hand Surgery Mission. Plast Surg Int. 2014;2014:921625.

25. Taylor KP, Ortiz A, Paltzer J. Short-term general, gynecologic, orthopedic, and pediatric surgical mission trips in Nicaragua: A cost-effectiveness analysis. J Glob Health. 2021;11:04024.

26. Shillcutt SD, Sanders DL, Teresa Butrón-Vila M, Kingsnorth AN. Cost-Effectiveness of Ingunial Hernia Surgery in Northwestern Ecuador. World J Surg. 2013 Jan 1;37(1):32–41.

27. Ament JD, Green K, Flores-Parra IV, Capobianco F, Otazo JC, Ramirez JC, et al. Health impact and economic analysis of NGO-supported neurosurgery in bolivia. Journal of Neurosurgery. 2011;115(2):A461.

28. Hamze H, Mengiste A, Carter J. The impact and cost-effectiveness of the Amref Health Africa-Smile Train Cleft Lip and Palate Surgical Repair Programme in Eastern and Central Africa. Pan African Medical Journal [Internet]. 2017 [cited 2024 Feb 19];28(1). Available from: https://www.ajol.info/index.php/pamj/article/view/167292

29. Egle JP, McKendrick A, Mittal VK, Sosa F. Short-term surgical mission to the Dominican Republic: A cost-benefit analysis. International Journal of Surgery. 2014 Oct 1;12(10):1045–9.

30. Schlegelmilch M, Rashiq S, Moreau B, Jarrín P, Tran B, Chuck A. Cost-Effectiveness Analysis of Total Hip Arthroplasty Performed by a Canadian Short-Stay Surgical Team in Ecuador. Advances in Orthopedics. 2017 Dec 18;2017:e5109895.

31. Eblovi D, Antúnez M, Clitheroe K, Meeks M, Balmert L, Thornton H, et al. Effectiveness, cost-effectiveness, and economic impact of a multi-specialty charitable surgical center in Honduras. International Journal of Surgery Open. 2019 Jan 1;20:7–14.

32. Hackenberg B. Measuring and Comparing the Cost-Effectiveness of Surgical C... : Journal of Craniofacial Surgery [Internet]. [cited 2024 Feb 19]. Available from: https://journals-lww-com.gate3.library.lse.ac.uk/jcraniofacialsurgery/abstract/2015/06000/measuring_and_comparing_the_cost_effectiveness_of.35.aspx
